# An Ultra‐Sensitive Quarantine Pathogen On‐Site Detection Based on a One‐Pot Asymmetric Recombinase Polymerase Amplification and MNAzyme‐Assisted Target Recycling Biosensor (OAR‐MNA)

**DOI:** 10.1111/pbi.70297

**Published:** 2025-08-14

**Authors:** Lei Yang, Guanwei Chen, Yongming Bo, Cheng Peng, Jiatong Yan, Xiaoyun Chen, Xiaoli Xu, Wei Wei, Xiaoxue Fang, Jian Wu, Xiaofu Wang, Meihao Sun, Junfeng Xu

**Affiliations:** ^1^ State Key Laboratory for Quality and Safety of Agro‐Products, Key Laboratory of Traceability for Agricultural Genetically Modified Organisms, Ministry of Agriculture and Rural Affairs, People’s Republic of China, Zhejiang Key Laboratory of Crop Germplasm Innovation and Utilization Zhejiang Academy of Agricultural Sciences Hangzhou China; ^2^ College of Life Sciences Zhejiang Normal University Jinhua China; ^3^ Key Laboratory of Watermelon and Cabbage Digital Seed Industry Ministry of Agriculture and Rural Affairs, Ningbo Weimeng Seed Industry Co. Ltd. Ningbo China; ^4^ College of Biosystems Engineering and Food Science Zhejiang University Hangzhou China

**Keywords:** asymmetric RPA, bacterial fruit blotch, cucumber green mottle mosaic virus, MNAzyme, one‐pot, ssDNA

## Abstract

The dissemination of quarantine pathogens poses a significant risk to global crop production, threatening crop health and disrupting agroecosystems. Timely and accurate on‐site detection is crucial to prevent outbreaks. Recombinase polymerase amplification (RPA) and multicomponent DNAzyme (MNAzyme) are promising isothermal detection technologies; however, their integration has been hindered by the requirement of single‐stranded DNA (ssDNA) for MNAzyme activation, as RPA generates double‐stranded DNA (dsDNA). To address this, we developed the OAR‐MNA biosensor, an innovative one‐pot platform coupling asymmetric RPA (aRPA) with MNAzyme‐assisted target recycling. The biosensor was applied to detect *Acidovorax citrulli* , the causative agent of bacterial fruit blotch (BFB) in cucurbits. By adjusting RPA primer concentrations, aRPA generates abundant ssDNA, enabling visual detection of *A. citrulli* with a detection limit of 20 copies/μL and perfect specificity, surpassing the performance of fluorescent RPA. The biosensor demonstrated 100% concordance with qPCR in seed samples and maintained robust performance in spiked melon homogenate. Our comparative analysis revealed advantages of the OAR‐MNA system, including reduced detection costs and enhanced field applicability. When coupled with a portable DNA analyser, the biosensor provides user‐friendly results (+/−), making it ideal for on‐site deployment. Beyond BFB detection, OAR‐MNA has been successfully applied to the detection of Cucumber green mottle mosaic virus (CGMMV), an RNA quarantine pathogen that causes severe losses to cucurbit crop production. By enabling early, accurate on‐site diagnosis, the OAR‐MNA biosensor represents an advancement in plant pathogen detection technology, offering a promising tool for safeguarding global crop production.

## 1 Introduction

Quarantine pathogens pose a serious threat to both global agriculture and natural ecosystems, causing substantial reductions in crop yield and quality worldwide (Rani et al. 2019). Among these pathogens, *Acidovorax citrulli* , the causative agent of bacterial fruit blotch (BFB), is particularly destructive to cucurbitaceous plants (Burdman and Walcott 2012; Martin and Horlock 2002). Classified as a quarantine pest, *A. citrulli* is listed as an agricultural phytosanitary threat in many countries. Despite its devastating impact, no effective means currently exist to prevent seed infection, highlighting the urgent need for accurate and timely detection methods to mitigate outbreaks and safeguard crop development.

Current standard for pathogen detection, including bacterial culturing, polymerase chain reaction (PCR) (Schrader et al. 2012; Yang and Rothman 2004), and enzyme‐linked immunosorbent assay (ELISA), is effective but relies on sophisticated equipment, lengthy protocols and trained personnel, limiting its applicability in resource‐limited settings. Isothermal amplification techniques, such as rolling circle amplification (RCA), loop‐mediated isothermal amplification (LAMP) and recombinase polymerase amplification (RPA), represent promising alternatives for pathogen diagnosis (Law et al. 2015; Notomi et al. 2000; Reid et al. 2018). These amplification techniques eliminate the need for thermocyclers while maintaining high sensitivity. Among these techniques, LAMP offers robustness against inhibitors but suffers from complex primer design requirements, which hinder its flexibility and broad adoption (Kim et al. 2023). RCA achieves high specificity at low temperatures, but the demand for additional enzymes and multistep workflows limits its suitability in on‐site detection (Wu et al. 2023). In contrast, RPA stands out for its rapid detection (< 30 min), tolerance to inhibitors (enabling direct analysis of crude plant extracts), and low operating temperature (37°C–42°C) (Craw and Balachandran 2012; Zhao et al. 2015). However, RPA has notable limitations, including nonspecific amplification (risk of false positives) and reliance on costly enzymes and fluorescent probes (multiple chemical modifications) (Daher et al. 2015; Morel et al. 1994; Munawar 2022).

RNA‐cleaving DNAzymes have emerged as versatile tools for pathogen detection (Santoro and Joyce 1997). Among these, multi‐component DNAzyme (MNAzyme) offers several advantages, including thermal stability, low cost, high catalytic efficiency and flexible design (Hanpanich et al. 2019; Mokany et al. 2010; Zhou et al. 2020). MNAzymes can be tailored to recognise any target sequence by modifying base pairing in their effector‐binding arms, offering a universal detection platform. Given that MNAzyme probes are approximately two‐fifths the cost of RPA probes, association with MNAzyme could reduce the cost of RPA‐based detection. Furthermore, this dual recognition approach, combining RPA with the MNAzyme system, makes it an ideal candidate for improving the detection specificity. However, integrating MNAzyme with RPA faces critical challenges: MNAzyme requires single‐stranded DNA (ssDNA) for activation, whereas RPA generates double‐stranded DNA (dsDNA).

To address this limitation, researchers have traditionally relied on DNA/RNA aptamers for MNAzyme‐based pathogen detection (Chang et al. 2021; Chen et al. 2021). However, aptamer selection via systematic evolution of ligands by exponential enrichment (SELEX) is labour‐intensive, time‐consuming, and complicates assay development (Yu et al. 2021). Alternative strategies, such as thermal denaturation (Yin et al. 2021) or chemical DNA denaturation (Hu et al. 2023; Mohamed et al. 2021), require open‐lid operations, increasing the risk of aerosol contamination.

In this study, we present an innovative one‐tube biosensor that combines asymmetric RPA (aRPA) with MNAzyme‐assisted assay (OAR‐MNA), enabling closed‐tube, contamination‐free pathogen detection. By employing a modified RPA protocol, our method efficiently generates abundant ssDNA to activate MNAzymes without additional processing steps. The MNAzyme probe functions around 41°C, making it compatible with both RPA and reverse transcription RPA (RT‐RPA) workflows. The dual‐recognition mechanism (RPA amplification followed by MNAzyme verification) ensures high specificity, while the cost‐efficient design reduces expenses by ~50% compared to fluorescent RPA (from $4.69 to $2.16 per reaction). Coupled with a pocket‐size DNA analyser that provides direct ‘+/−’ outputs, the OAR‐MNA biosensor achieves field‐deployable detection of *A. citrulli* . Furthermore, to demonstrate broad applicability across pathogen types, we adapted the platform for detection of Cucumber green mottle mosaic virus (CGMMV), a globally significant RNA quarantine pathogen (Dombrovsky et al. 2017), requiring only substitution of RPA with RT‐RPA. Together, our work provides a cost‐effective, user‐friendly and adaptable solution for on‐site diagnosis, mitigating the risks of pathogen transmission through infected seeds. This advancement offers a critical tool for a broad range of plant pathogens and ultimately improves the safety and health of agricultural production systems.

## 2 Materials and Methods

### 2.1 Reagents and Materials

The details of reagents and materials used in this work were supplied in Supporting Information. The sequences of the primers, partzymes (containing split catalytic domain of DNAzyme 10–23), synthetic ssDNA target, and probes were listed in Table S1.

### 2.2 Polyacrylamide Gel Electrophoresis and Fluorescent Intensity Measurement for Assay Feasibility Analysis

To validate the cleavage capability of the MNAzyme, each well was loaded with a total volume of 10 μL reaction assay, using the following concentrations: 500 nM AC‐P A, 500 nM AC‐P B, 1250 nM MNA fluorescence quencher (FQ)‐probe, 500 nM AC‐ssDNA and MNAzyme buffer containing 100 mM MgCl_2_. The feasibility of the OAR‐MNA biosensor for target nucleic acid detection was further examined by both the gel electrophoresis and the fluorescent intensity. RPA or aRPA products incubated with AC‐P A/B complexes with or without probes were recorded. The standard RPA assay was conducted in accordance with the manufacturer's instructions (see Supporting Information for details). For aRPA, we employed the optimised primer ratio of 10 000 nM (forward) to 31.25 nM (reverse) as determined through systematic evaluation (see Supporting Information for details). Post‐amplification, reaction products were analysed by combining 10 μL of either RPA or aRPA amplicons with 40 μL of MNAzyme reaction and incubated for 15 min. The products were conducted using 15% polyacrylamide gel electrophoresis (PAGE) at a constant voltage of 120 V for 40 min. The fluorescence intensity of the RPA or aRPA products combined with the MNAzyme assay was also measured.

### 2.3 Development of an OAR‐MNA Biosensor

**FIGURE 1:**
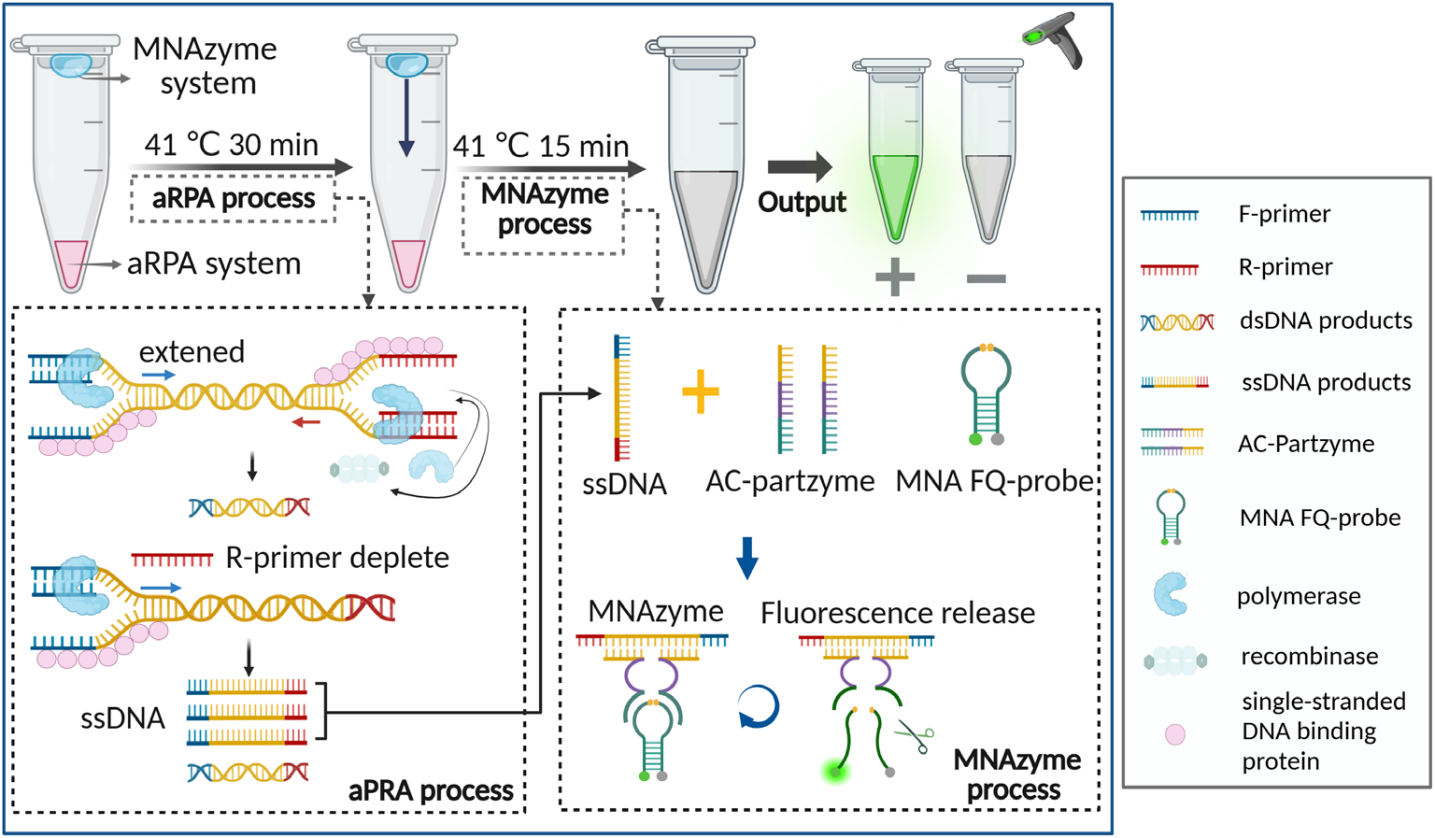
Schematic illustration of the OAR‐MNA biosensor signal amplification. Bottom layer labelled as aRPA system, containing DNA template, F/R‐primers, enzymes and buffers. Lid layer labelled as MNAzyme system, comprising partzymes, MNA fluorescence quencher (FQ) probe, Mg^2+^ and buffers. Step 1 (aRPA process): Illustrates dsDNA amplification (blue/red arrows), followed by ssDNA production (blue arrow only) upon depletion of R‐primers. Step 2 (MNAzyme process): Shows centrifugation merging lid layers and bottom layers, with ssDNA hybridising to partzymes to form active MNAzymes. These activated MNAzymes then continuously cleave the RNA‐containing FQ probes, generating an amplified fluorescent signal.

The OAR‐MNA biosensor consisted of two separate RPA and MNAzyme components. The RPA included 6 μL of rehydration A buffer, 0.4 μL of each RPA primer at various ratios, 1.7 μL double distilled water, the recommended dose of recombinase and polymerase, and 0.5 μL B‐buffer containing MgOAc. The MNAzyme premix was used according to the recommended concentrations from the ssDNA‐based MNAzyme assay (see Supporting Information for details). The RPA was added to the bottom of the tubes and the MNAzyme component to the lid of the tubes. Following a 30‐min aRPA process, the tubes were briefly centrifuged to introduce the MNAzyme components into the reaction mixture, and the assay was continued for an additional 15 min to allow for MNAzyme assembly and probe cleavage (Figure 1). Optimization experiments focused on the precise temperature range, Mg^2+^ concentration, AC‐P A/B concentration, MNA FQ‐probe concentration, aRPA/MNAzyme volume ratio, and aRPA primer ratio (See Supporting Information for details).

### 2.4 Assay Sensitivity and Specificity Evaluation

The sensitivity of the OAR‐MNA biosensor was assessed by testing serial dilutions of the *A. citrulli* plasimid‐T (ranging from 2 × 10^0^ to 2 × 10^6^ copies/μL), with double‐distilled water as the non‐template control (NTC). To evaluate specificity, we examined the potential cross‐reactivity of the related pathogen strains, with the RPA primers (AC‐SP‐2/AC‐AP‐2) specific to *A. citrulli* . Genomic DNA extracted from these pathogen strains served as templates for the test.

To establish comparative performance benchmarks, specificity and sensitivity assessments were conducted against the published RPA method for BFB detection (Wang et al. 2022), utilising identical primers and probes (Ac‐F3, Ac‐R2, Ac‐P) used in their study.

Endpoint signals from all experiments were visualised and integrated into the corresponding figures, demonstrating the viability of the OAR‐MNA biosensor and fluorescent RPA in terms of visualisation.

### 2.5 Construction of a Pocket‐Size DNA Analyser

The shell of the analyser was designed using SolidWorks software, with final dimensions of 74 × 74 × 76 mm. The analyser integrated two critical functions: temperature control and fluorescence signal output. These functions were achieved through the following main modules: (1) Temperature Control Module, a thermostatic control system with both heating and cooling capabilities. (2) Laser Light Source Module, incorporating a 480 nm laser light for fluorescence excitation. (3) Signal Detection Module, including a fluorescent signal receiver and a main control board that translates the fluorescence signal into an electrical signal, which is then transmitted to the display screen and output as ‘+’ or ‘−’.

The pocket‐size DNA analyser was used to set the positive threshold value as the mean plus three standardised deviations (mean + 3 SD) (Gao et al. 2024; Yu et al. 2023) from randomly chosen negative controls (NC). According to preset positive threshold, the output was ‘+’ if the value exceeded the threshold and ‘−’ if it did not.

### 2.6 Statistical Analysis

To ensure data accuracy, all experiments were conducted in triplicate. Statistical significance was analysed using one‐way analysis of variance (ANOVA) followed by Duncan's multiple‐range test (*p < 0.05, **p < 0.01, ***p < 0.001, ****p < 0.0001). The threshold was established at the 95% confidence level, computed as mean + 3 SD from randomly chosen NC. All graphical plots presented in this study were generated and analysed by using Origin 10.0 software.

## 3 Results and Discussion

### 3.1 Illustration of the OAR‐MNA Biosensor

We have developed a novel, rapid, simple and sensitive platform for the detection of BFB. The OAR‐MNA biosensor workflow was illustrated in Figure 1 and could be divided into three steps: aRPA assay, post‐added MNAzyme assay and result readout. (1) aRPA assay: during the aRPA process, numerous ssDNA fragments are obtained just by adjusting the concentration of RPA F‐primer (excess) and R‐primer (restricted). As shown in Figure 1, RPA forward and reverse primers bind to the DNA template and produce numerous dsDNA (Figure 1). As the R‐primers are depleted, F‐primers bind to the RPA‐produced dsDNA and generate abundant ssDNA (Kober et al. 2018; Zhang et al. 2025). (2) Post‐added MNAzyme assay: the MNAzyme activates through structural assembly upon hybridization with ssDNA amplicons, utilising FQ probes containing specific RNA cleavage sites (Figure S1). (3) Sensitive signal readout: activated MNAzymes exhibit immediate catalytic activity, selectively cleaving the MNA FQ‐probe and releasing green fluorescence detectable by three methods: visual inspection under UV light, real‐time PCR detection or portable DNA analyser measurement (Figure S2).

### 3.2 Gel Analysis of Asymmetric RPA Products

Using the AC‐SP‐2 and AC‐AP‐2 primer pair (Figure S3), we systematically optimised primer concentration to maximise ssDNA yield. Our electrophoretic analysis (Figure S4) revealed that as the ratio decreased from 1:1 to 320:1, the intensity of the ~160 bp RPA amplicon band (the correct AC‐SP‐2/AC‐AP‐2 amplified band) diminished, while lower and upper bands became more prominent. Previous studies have identified the lower band as ssDNA comprising the sense strand of the RPA amplified fragment, and the upper band as a hybrid dsDNA structure (Yang et al. 2024). Based on the lower band intensity, we determined optimal production at primer ratios between 160:1 and 320:1. Subsequent evaluation across 2–2 × 10^6^ copies/μL template concentrations utilised a primer ratio of 320:1 (10 000 nM:31.25 nM) to establish the system's dynamic performance characteristics. We found reliable ssDNA generation from 200 copies/μL onward, with yield showing a positive correlation to input DNA (Figure S5). These results confirm the robust performance of our aRPA system in generating visualisable ssDNA products across a wide concentration range, while establishing optimal primer conditions for downstream MNAzyme detection. The clear electrophoretic patterns and consistent yield trends confirm the suitability of this amplification approach for analysing field samples with varying bacterial loads, making it particularly valuable for agricultural diagnostics.

### 3.3 The Feasibility of the OAR‐MNA Biosensor

**FIGURE 2:**
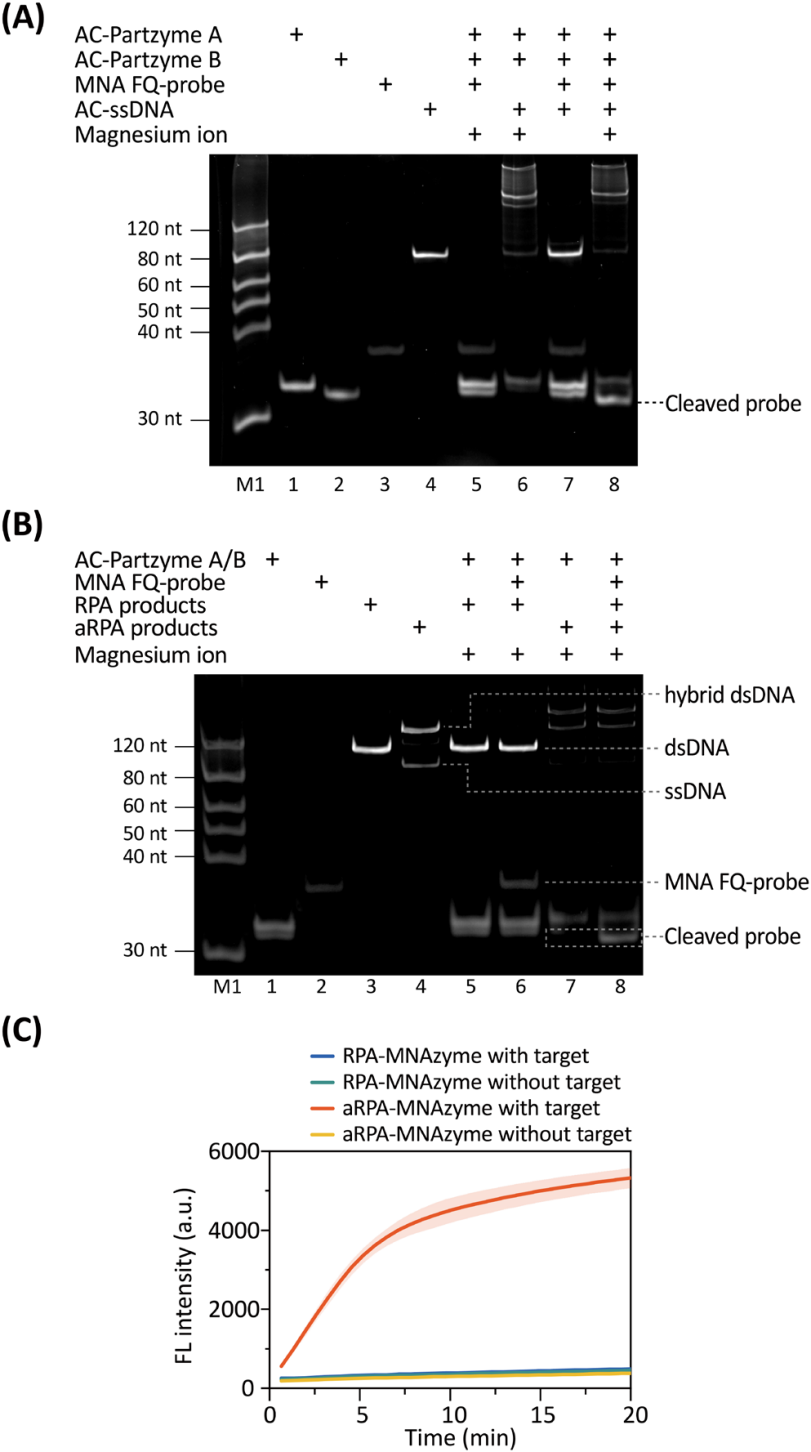
Feasibility of the OAR‐MNA biosensor. (A) PAGE analysis confirming MNAzyme‐mediated FQ‐probe cleavage using 500 nM synthetic *A. citrulli* ssDNA (Ac‐ssDNA). (B) PAGE analysis of MNA FQ‐probe cleavage with RPA (dsDNA) versus aRPA (ssDNA) products. Main bands are illustrated. Template concentration: 2 × 10^3^ copies/μL. (C) Fluorescence kinetic analysis of the OAR‐MNA biosensor during RPA or aRPA processes with or without target. Template concentration: 2 × 10^6^ copies/μL.

The feasibility of the MNAzyme assay was initially verified using gel electropherogram. As shown in Figure 2A, lane 8 demonstrated the diminishment of the AC‐ssDNA band and the emergence of a new band, indicating MNAzyme conformational rearrangement and probe cleavage. As the MNAzyme 10–23 is a DNA metalloenzyme requiring divalent metal ions for RNA transesterification (Kim et al. 2007; Wang et al. 2011), we examined magnesium's catalytic role. The absence of cleavage in Mg^2+^‐free assay (lane 7) versus cleavage with Mg^2+^ assay (lane 8) clearly demonstrated the essential cofactor function of magnesium in enabling MNAzyme activity.

To clarify the interaction between RPA/aRPA products and MNAzyme, we substituted AC‐ssDNA with RPA and aRPA products. A DNA template concentration of 2 × 10^3^ copies/μL was used for gel electrophoresis. Figure 2B demonstrates that RPA products (lane 6) failed to induce probe cleavage, evidenced by intact FQ‐probe bands. In contrast, the addition of the MNAzyme reaction led to the degradation of the ssDNA band and the appearance of a new band (lanes 7 and 8), confirming aRPA‐generated ssDNA activates MNAzyme (Figure 2B). Fluorescence kinetics (Figure 2C) confirmed exclusive signal generation with aRPA products, validating ssDNA‐dependent MNAzyme activation. Even under high template abundance (2 × 10^6^ copies/μL), direct RPA products (dsDNA) failed to elicit detectable signals when combined with MNAzyme. Collectively, these results demonstrate the feasibility of the aRPA and MNAzyme combined assay, showcasing the potential of the developed biosensor as a powerful tool for detection applications.

### 3.4 Establishment of the OAR‐MNA Biosensor

**FIGURE 3:**
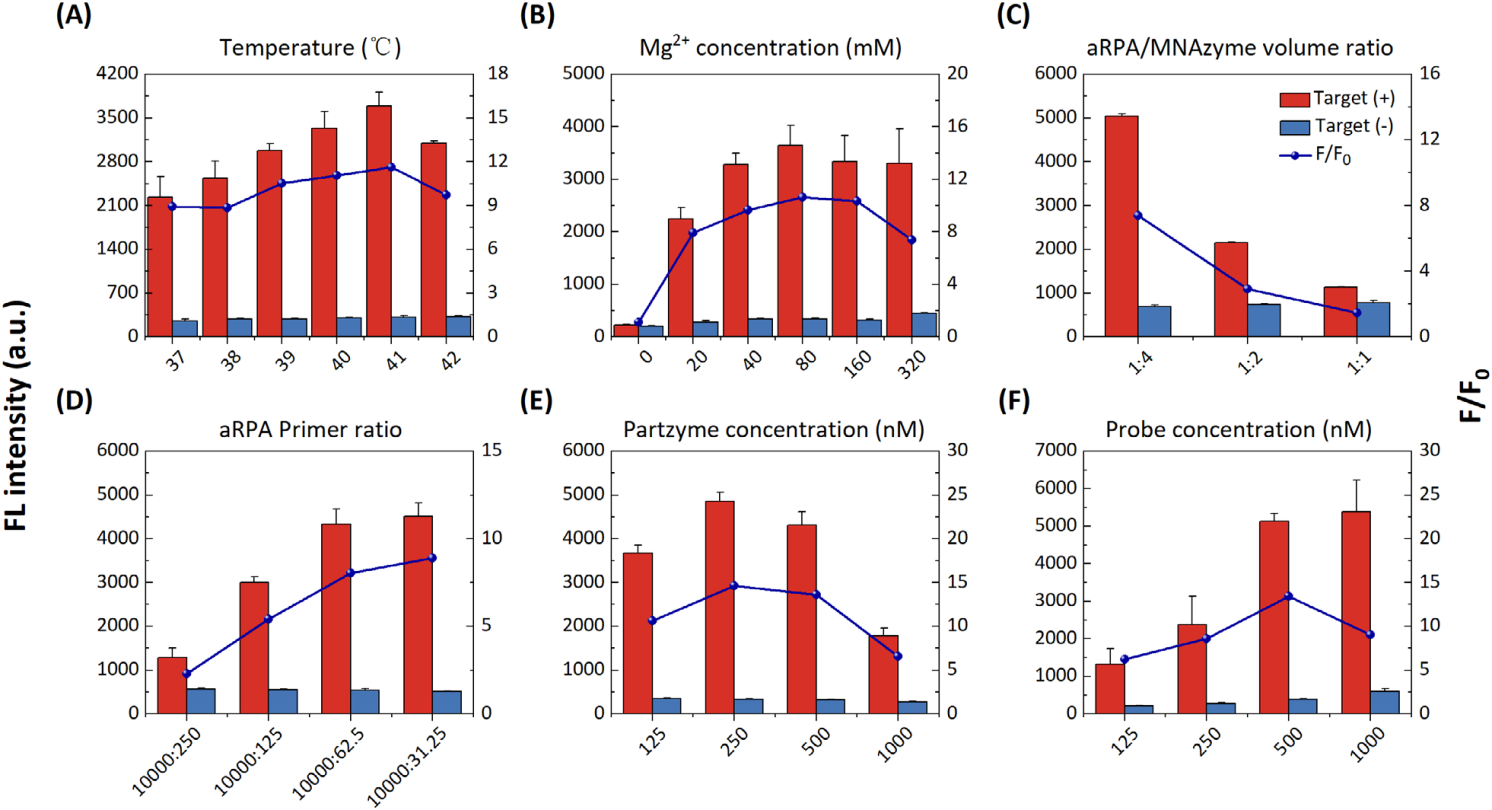
Optimising OAR‐MNA biosensor reaction conditions. (A) Selection of optimal working temperature, (B) Mg^2+^ concentration, (C) volume ratio of aRPA and MNAzyme, (D) aRPA primer ratio, (E) partzyme concentration and (F) probe concentration. Error bars reflect means ± standard deviation from triplicate experiments (*F*: FL intensity of the corresponding conditions with target; *F*:_0_: blank FL intensity of the corresponding conditions without target).

The details of AC‐partzyme screening and optimisation of ssDNA‐based MNAzyme reaction parameters (Figures S6–S8) were provided in the Supporting Information. To establish the OAR‐MNA biosensor, reaction conditions were systematically optimised for temperature, reagent concentrations and reaction volume ratios. As shown in Figure 3A and Figure S9A, fluorescence intensity and signal‐to‐noise ratio (*F*/*F*_0_) initially increased and then decreased, with the optimal temperature at 41°C. Similarly, fluorescence increased with Mg^2+^ concentrations, reaching a maximum at 80 mM (Figure 3B and Figure S9B), consistent with reported optimal MNAzyme activity at 40–80 mM Mg^2+^ (Kang et al. 2021; Larraga‐Urdaz et al. 2024; Liu et al. 2022). The aRPA:MNAzyme volume ratio showed an inverse relationship with fluorescence intensity, with optimal performance at 1:4 (Figure 3C and Figure S9C). Primer ratio optimisation confirmed enhanced signals with decreasing reverse primer concentrations (Figure 3D and Figure S9D). We selected 10 000 nM:31.25 nM, within the literature‐established range for maximal ssDNA yield (31.25–93 nM) (Bemetz et al. 2019; Elsaesser et al. 2018; Yang et al. 2024). AC‐P A3/B3 and probe concentrations were optimised at 250 nM (Figure 3E and Figure S9E) and 500 nM (Figure 3F and Figure S9F), respectively.

### 3.5 Performance Assessment of the OAR‐MNA Biosensor

**FIGURE 4:**
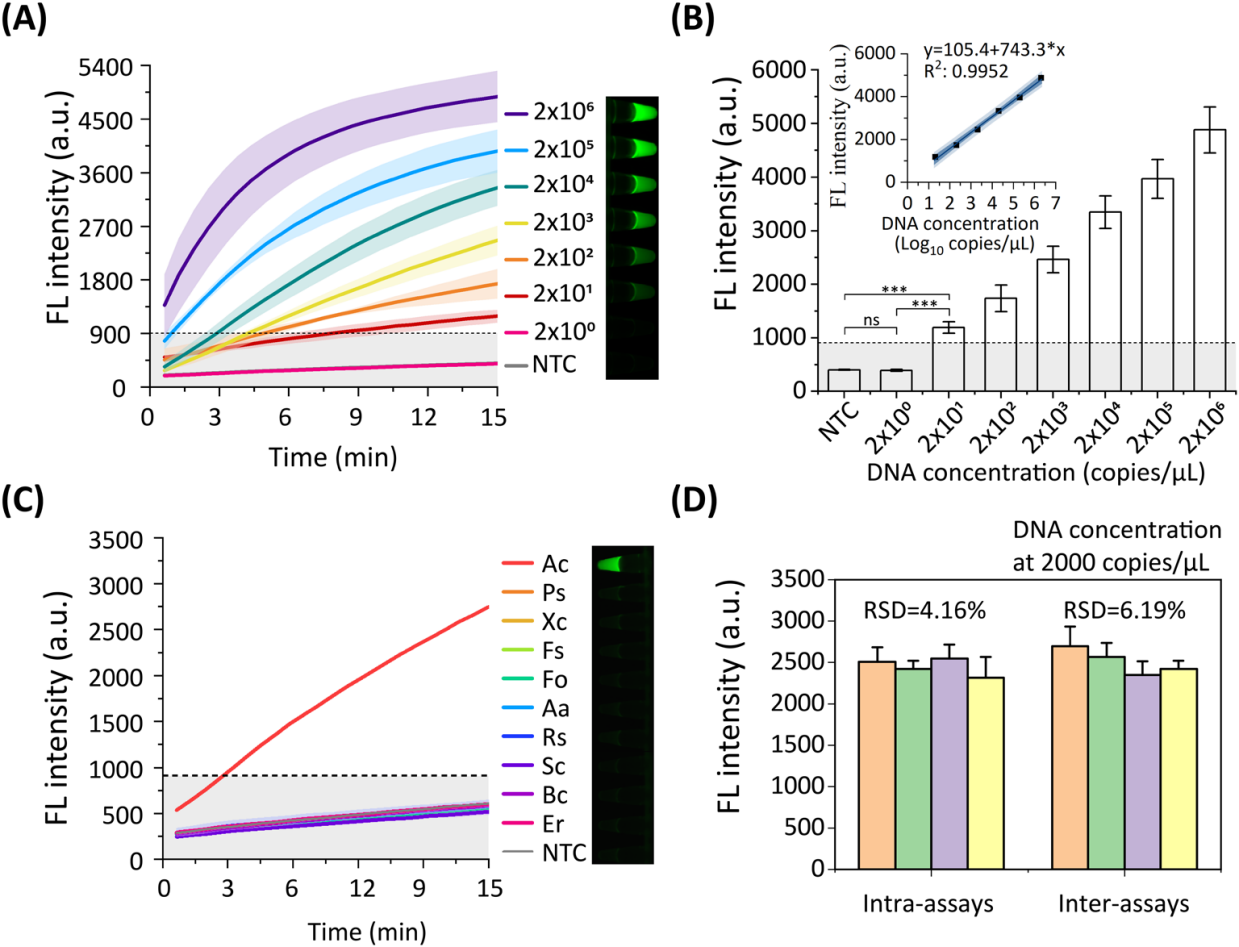
Sensitivity and specificity assessment of the OAR‐MNA biosensor. (A) Real‐time detection curves and fluorescence images at various plasmid‐T DNA concentrations. (B) Bar charts document the OAR‐MNA biosensor endpoint signals. Standard curve plot generated from the results of the OAR‐MNA biosensor, displaying the slope, *Y*‐intercept and *R* ^2^ value. (C) Real‐time detection curves and fluorescence images among 10 related pathogen species. All pathogen genomic DNAs were adjusted to 0.1 ng/μL prior to the experiment. Aa, *Acidovorax avenae* subsp. *avenae*; Ac, *Acidovorax citrulli* ; Bc, *Botrytis cinerea*; Er, *Exserohilum rostratum*; Fo, *Fusarium oxysporum*; Fs, *Fusarium solani*; Ps, *Pseudomonas syringae* ; Rs, *Ralstonia solanacearum* ; Sc, *Stagonosporopsis cucurbitacearum*; Xc, *Xanthomonas cucurbita*. (D) Intra‐assay and inter‐assay results of the OAR‐MNA biosensor by using 2000 copies/μL plasmid DNA. Fluorescent images in (A) and (C) were taken at the endpoint of the detection curves. Error bars represent means ± standard deviation from triplicate experiments. Asterisks denote statistical significance (*p < 0.05, **p < 0.01, ***p < 0.001, ****p < 0.0001). NTC represents non‐template control. The horizontal dashed line indicates the threshold fluorescence intensity.

Our systematic evaluation demonstrated the OAR‐MNA biosensor's excellent performance characteristics for pathogen detection. Sensitivity testing using serial dilutions of *A. citrulli* DNA revealed a detection limit of 20 copies/μL, based on both real‐time detection curves and fluorescence images (Figure 4A). The biosensor showed excellent linearity (*R* ^2^ > 0.99; Figure 4B), indicating its suitability for quantitative assessments (Wang et al. 2024). Intra‐assay and inter‐assay relative standard deviations (RSDs) were calculated at 4.16% and 6.19%, respectively (Figure 4D), demonstrating the method's good repeatability and reproducibility. Specificity assessment against 10 related pathogens confirmed exclusive detection of *A. citrulli* without cross‐reactivity (Figure 4C and Figure S10).

Comparative analysis with fluorescent RPA revealed significant advantages of our biosensor. While both methods theoretically detect 20 copies/μL, the OAR‐MNA biosensor achieved reliable visual detection at this threshold, whereas fluorescent RPA showed marginally visual discrimination at this level (Figure 4A and Figure S11). Moreover, OAR‐MNA exhibited no cross‐reactivity with *A. avenae* subsp. *avenae*, whereas RPA yielded false positives (Figure 4C and Figure S12). Gel electrophoresis (Figure S13) confirmed amplification of both *A. citrulli* and *A. avenae* subsp. *avenae* DNA, yielding expected ~160 bp products. Detailed sequence alignment results and fluorescence kinetics analysis (Figures S14–S19) elucidated the molecular basis for RPA cross‐reactivity and the maintained specificity of our MNAzyme‐based system (see Supporting Information for details). Notably, our approach offers a ~50% reduction in per‐reaction costs, despite a modestly longer assay time.

These results validate OAR‐MNA as a superior diagnostic tool combining slightly enhanced sensitivity (20 copies/μL visual detection), perfect specificity, and cost‐effectiveness for field applications.

### 3.6 Real Sample Detection by the OAR‐MNA Biosensor

**FIGURE 5:**
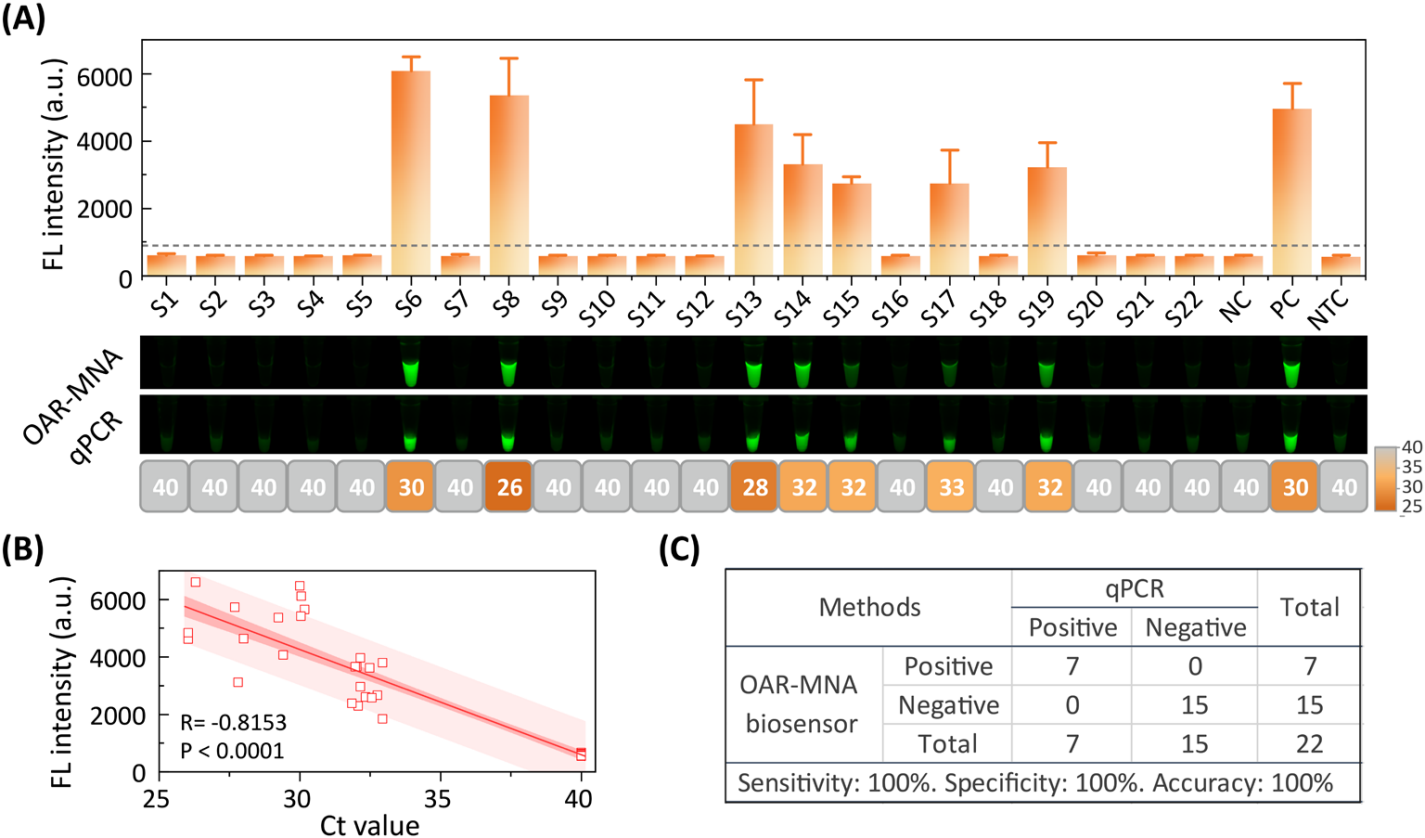
Robust performance of the OAR‐MNA biosensor for BFB detection in watermelon seeds. (A) Comparison of OAR‐MNA biosensor and qPCR results for 22 watermelon samples. Endpoint OAR‐MNA biosensor results are depicted as bar charts. Error bars represent means ± standard deviation from three technical triplicates. NC, negative control; NTC, non‐template control; PC, positive control. Horizontal dashed line indicates threshold fluorescence intensity. Orange rectangle boxes represent positive signals in the qPCR assay, while grey rectangle boxes indicate negative results. Fluorescent images by OAR‐MNA biosensor and qPCR are also included. (B) Correlation between parallel measurements of *A. citrulli* using the OAR‐MNA biosensor (fluorescence intensity, *y*‐axis) and qPCR (*Ct* values, *x*‐axis). Linear regression was performed to generate a linear correlation curve. (C) Performance characteristics of the OAR‐MNA biosensor.

Biological samples, particularly plant tissues and seeds, contain inhibitors that can interfere with nucleic acid amplification. Polysaccharides (e.g., pectin), secondary metabolites and polyphenolics (including tannic acid) are primary inhibitors that can form complexes with nucleic acids or inhibit DNA polymerase activity (Suther and Moore 2019; Koonjul et al. 1999; Thompson et al. 2014). To validate practical applicability, we tested 22 watermelon seed samples potentially contaminated with *A. citrulli* , comparing results against qPCR as the gold standard. The OAR‐MNA biosensor identified *A. citrulli* in seven samples, showing perfect concordance with qPCR (Figure 5A; Figures S20 and S21). Quantitative analysis revealed a strong correlation between methods (Pearson's *r* = −0.8153, Figure 5B), with equivalent diagnostic accuracy (Figure 5C).

The matrix effect of melon homogenates was not clear. To investigate this, we conducted a comparable detection on pure *A. citrulli* colonies and watermelon homogenates spiked with *A. citrulli* (Table S2). While qPCR showed minimal impact from interference substances, our OAR‐MNA biosensor demonstrated reliable performance in complex matrices. Although quantitative differences occurred between pure colonies and spiked samples (likely due to matrix effects), the OAR‐MNA biosensor successfully detected *A. citrulli* across high, medium and low concentration ranges in spiked samples. These results confirm that our biosensor maintains sensitivity in complex matrices, supporting practical implementation potential for agricultural biosecurity.

To demonstrate the broad applicability of OAR‐MNA biosensor across pathogen types, the system was applied to CGMMV detection by replacing RPA assay with RT‐RPA assay (see Supporting Information for details). We selected optimal RPA primers and partzymes (Figures S22 and S23) to develop an effective OAR‐MNA biosensor for CGMMV. The system exhibited exclusive CGMMV specificity, generating robust fluorescence signals without cross‐reactivity to other plant viruses (Figure S24). Comparative evaluation using watermelon seed samples demonstrated full concordance between OAR‐MNA and qPCR method (Figures S25 and S26). These data further emphasise the versatility of OAR‐MNA for diverse quarantine pathogens.

### 3.7 Development of a Pocket‐Size DNA Analyser

**FIGURE 6:**
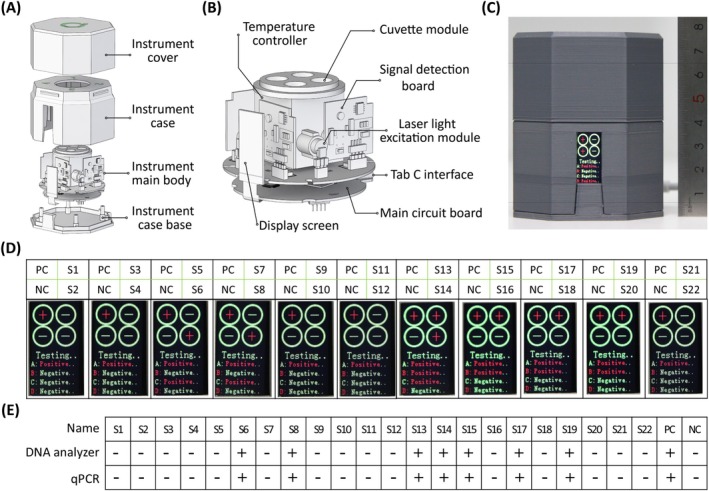
Construction and results analysis of the pocket‐size DNA analyser. (A) Exploded view of the pocket‐size DNA analyser. (B) Detailed composition of the main body of the analyser. (C) Images of the prototype of the pocket‐size DNA analyser. (D) Results of 22 watermelon seed field samples analysed using the DNA analyser. Each test can detect two samples, with both positive and negative samples included as controls. NC denotes negative control, and PC denotes the positive control. (E) Field sample detection comparing OAR‐MNA biosensor using the pocket‐size DNA analyser and qPCR assays using the Bio‐Rad CFX96 system.

Given the OAR‐MNA biosensor's requirement for a constant temperature of approximately 41°C, we developed a compact, power bank‐powered, pocket‐size DNA analyser equipped with a semiconductor temperature controller to maintain the necessary reaction conditions (Figure 6). The analyser features a 480 nm laser light‐excitation module to excite the FAM‐tagged probe. The laser light source and fluorescent signal collection module were positioned at 90°, with a dichroic mirror used to change the excitation light path (Figure 6B). The main control board converts the fluorescent signals into direct ‘+’ (positive) or ‘−’ (negative) output, thereby enabling immediate interpretation. The feasibility of the pocket‐size DNA analyser was validated by testing the same 22 watermelon seed samples in Figure 5. The analyser yielded results identical to those obtained using qPCR (Figure 6D,E). Owing to the portability of the DNA analyser, the OAR‐MNA biosensor presents its efficacy in on‐site testing, particularly in resource‐limited regions.

### 3.8 Comparative Analysis and Practical Applications of the OAR‐MNA Biosensor

Through systematic comparison with existing isothermal amplification methods and PCR‐based assays, we evaluated the OAR‐MNA biosensor's working principles, advantages, limitations and cost‐effectiveness (Table S3). Additionally, we assessed the practical performance characteristics of these methods, including sensitivity, specificity and operational requirements (Table S4).

Our analysis reveals several notable advantages of the OAR‐MNA biosensor relative to conventional diagnostic approaches. The system achieves laboratory‐comparable sensitivity (20 copies/μL) and specificity within 45 min while offering five key benefits for resource‐constrained environments: (1) enhanced reagent stability at room temperature (maintaining activity for days without refrigeration); (2) simplified detection using our purpose‐built portable analyser that provides unambiguous readouts (+/−); (3) minimal infrastructure demands, requiring only basic micropipettes and consumables (Figure S27); (4) straightforward two‐step protocol requiring minimal technical training; and (5) superior cost‐efficiency at $2.16 per test. Field evaluations demonstrated perfect concordance (100%) with qPCR benchmarks, validating its reliability for agricultural biosecurity applications.

While the current system is limited to single‐target detection per tube, further research will focus on multiplexed detection. Its combination of analytical performance, operational simplicity and cost efficiency makes it particularly advantageous for routine plant pathogen monitoring in developing regions where resources are limited.

## 4 Conclusions

Overall, the developed OAR‐MNA biosensor possesses the ability to detect *A. citrulli* with high sensitivity (20 copies/μL), specificity, and quantitative capability (*R* ^2^ > 0.99). Notably, its applicability was successfully extended to CGMMV detection, demonstrating feasibility for different quarantine pathogens. The biosensor achieved several key innovations: (1) an ssDNA‐based detection system eliminating the need for DNA/RNA aptamers, (2) a contamination‐free, one‐pot assay format, and (3) integration with portable instrumentation for field‐deployable diagnostics. Comparative studies demonstrate superior performance to fluorescent RPA methods, particularly in specificity and visual sensitivity.

While the current platform shows great promise, we recognise two important limitations for future improvement: (1) single‐target detection capacity; and (2) dependence on standard DNA extraction protocols. Future research will address these challenges by: (1) developing multiplexed detection capabilities for simultaneous detection of various pathogens; and (2) adopting an extraction‐free method to simplify the DNA extraction procedure. The OAR‐MNA platform effectively addresses the critical need for field‐deployable diagnostics, balancing laboratory‐grade accuracy with practical features tailored for challenging environments. These improvements will further enhance the biosensor's applicability to a wider range of plant pathogens and could contribute to more effective disease management strategies in agriculture, particularly in resource‐constrained environments.

## Author Contributions

**Lei Yang:** conceptualization, data curation, formal analysis, funding acquisition, methodology, project administration, software, writing – original draft. **Guanwei Chen:** data curation, formal analysis, investigation, methodology, software. **Yongming Bo:** methodology, investigation. **Cheng Peng:** methodology, conceptualization. **Jiatong Yan:** methodology, investigation. **Xiaoyun Chen:** writing – review and editing. **Xiaoli Xu:** writing – review and editing. **Wei Wei:** writing – review and editing. **Xiaoxue Fang:** methodology, investigation. **Jian Wu:** writing – review and editing. **Xiaofu Wang:** writing – review and editing, conceptualization, methodology, validation. **Meihao Sun:** writing – review and editing, methodology, validation. **Junfeng Xu:** conceptualization, writing – review and editing, supervision, funding acquisition.

## Conflicts of Interest

The authors declare no conflicts of interest.

## Supporting information


**Data S1:** pbi70297‐sup‐0001‐Supinfo.docx.

## Data Availability

The data that supports the findings of this study are available in the Supporting Information of this article.
