# Supplementary material for: An Ultra‐Sensitive Quarantine Pathogen On‐Site Detection Based on a One‐Pot Asymmetric Recombinase Polymerase Amplification and MNAzyme‐Assisted Target Recycling Biosensor (OAR‐MNA)
Source: Plant Biotechnol J. 2025 Aug 14;23(12):5387–96. doi: 10.1111/pbi.70297 (PMC12665056; doi:10.1111/pbi.70297)
Supplement: Supplementary file 1 — Data S1: pbi70297‐sup‐0001‐Supinfo.docx. [file PBI-23-5387-s001.docx]

**Supplementary Material**

**An ultra-sensitive quarantine pathogen on-site detection based on a one-pot asymmetric recombinase polymerase amplification and MNAzyme-assisted target recycling biosensor (OAR-MNA)**

Lei Yang^1, b^, Guanwei Chen^1, a^, Yongming Bo^c^, Cheng Peng^b^, Jiatong Yan^a^, Xiaoyun Chen^b^, Xiaoli Xu^b^, Wei Wei^b^, Xiaoxue Fang^c^, Jian Wu^d^, Xiaofu Wang^*, b^, Meihao Sun^*, a^, and Junfeng Xu^*, b^

^a^ College of Life Sciences, Zhejiang Normal University, Jinhua 321004, China.

^b^ State Key Laboratory for Quality and Safety of Agro-products, Key Laboratory of Traceability for Agricultural Genetically Modified Organisms, Ministry of Agriculture and Rural Affairs, P.R.China, Zhejiang Key Laboratory of Crop Germplasm Innovation and Utilization, Zhejiang Academy of Agricultural Sciences, Hangzhou 310021, China

^c^Key Laboratory of Watermelon and Cabbage Digital Seed Industry,Ministry of Agriculture and Rural Affairs, Ningbo Weimeng Seed Industry Co., Ltd, Ningbo 315101, China

^d^College of Biosystems Engineering and Food Science, Zhejiang University, Hangzhou 310058, China

*Correspondence: yywxf1981@163.com; mhsun@zjnu.cn; njjfxu@163.com

^1^These authors contributed equally to this work.

**Table of Contents**

1. **Experimental**

1.1. Reagents and materials

1.2. RPA assay procedures

1.3 AC-Partzyme screening and ssDNA-based MNAzyme assay optimization

1.4 Optimization of the OAR-MNA biosensor

1.5 Molecular mechanism analysis of OAR-MNA specificity and fluorescent RPA cross-reactivity

1.6 Performance of OAR-MNA biosensor on real seed samples

1.7 Performance of OAR-MNA biosensor on spiked watermelon homogenate samples

1.8. qPCR toward BFB detection

1.9 Application of OAR-MNA in Cucumber green mottle mosaic virus (CGMMV) detection

1. **Supplementary Figures and Tables**

Figure S1. Schematic illustration of the specific designs of the base sequences of MNA FQ-probe, partzyme A/B and target ssDNA for triggering the cleavage reaction.

Figure S2. The result output mode of the OAR-MNA biosensor.

Figure S3. Electrophoresis of RPA amplicon using *Acidovorax citrulli* specific RPA primers (AC-SP-2/AC-AP-2) with or without targets.

**Figure S4.** Primer ratio optimization for aRPA.

**Figure S5.** Dynamic range assessment of ssDNA yield by testing template concentrations across a wide range (2-2×10⁶ copies/μL) using a primer ratio of 320 (10,000 nM) :1 (31.25 nM).

**Figure S6.** Real-time curves of the ssDNA-based MNAzyme with different reaction conditions.
**Figure S7.** Optimizing ssDNA-based MNAzyme reaction conditions.

**Figure S8.** Stability of MNAzyme reagents on 8-strip PCR tube lid.

**Figure S9.** Real-time curves of the OAR-MNA biosensor with fine-tuned key parameters.

**Figure S10.** OAR-MNA biosensor specificity for BFB detection.

**Figure S11.** Sensitivity detection of *Acidovorax* *citrulli* via fluorescent RPA assay.

**Figure S12.** Specificity assessment of *Acidovorax* *citrulli* via fluorescent RPA assay.

**Figure S13.** Gel electrophoresis of RPA amplicons among ten related pathogen species using Ac-F3/Ac-R2 primer set.

**Figure S14.** Sequence alignment of Ac-F3/Ac-R2 RPA amplicons.

**Figure S15.** Electrophoretic comparison of RPA and aRPA products.

**Figure S16.** Sense-strand sequence alignment of aRPA amplicons.

**Figure S17.** Antisense-strand sequence alignment highlighting MNAzyme target sites.

**Figure S18.** Additional antisense-strand sequence alignment of *Acidovorax avenae* subsp. *avenae* aRPA amplicons.

**Figure S19.** MNAzyme activation kinetics with synthetic ssDNA.

**Figure S20.** Heatmap of endpoint fluorescent signals of 22 watermelon seeds detected by the OAR-MNA biosensor.

**Figure S21.** Real-time curves of the qPCR results for 22 watermelon seeds.

**Figure S22** Electrophoresis of RT-RPA amplicon with CGMMV RPA primer pairs.

**Figure S23.** Optimization of CGMMV-specific partzyme pairs.

**Figure S24.** Specificity validation of OAR-MNA for CGMMV detection.

**Figure S25**. Field detection of CGMMV in watermelon seeds using OAR-MNA.

**Figure S26**. qPCR validation of CGMMV in watermelon seed samples.

**Figure S27.** Detection of samples by the OAR-MNA biosensor using a DNA analyzer in a resource-limited field environment.

**Table S1.** Sequences of primers and probes used in this work.

**Table S2.** Comparison of fluorescent intensities between pure *Acidovorax citrulli* cultures and *A. citrulli*-spiked watermelon homogenates at different concentrations.

**Table S3.** Comparison of the working principle, key advantages, disadvantages and costs between the most widely used nucleic acid-based detection methods in plant disease detection and our established method.

**Table S4.** Comparison of the sensitivity, specificity and procedures between the most widely used nucleic acid-based detection methods in plant disease detection and our established method.

1. **References**
2. **Experimental section**
   1. **Reagents and materials**

Bacterial strains *Acidovorax citrulli* (ATCC 29625), *Xanthomonas cucurbita*, *Acidovorax avenae* subsp. *avenae*, and *Ralstonia solanacearum* were obtained from the China General Microbiological Culture Collection Center (CGMCC) and BeNa Culture Collection. Six additional melon pathogens (*Pseudomonas syringae*, *Fusarium solani*, *Fusarium oxysporum*, *Stagonosporopsis cucurbitacearum*, *Botrytis cinerea*, and *Exserohilum rostratum*), were isolated from infected melon tissues and maintained in our laboratory. Bacterial strains (*A. citrulli*, *A. avenae* subsp. *avenae*, *R. solanacearum*, *P. lachrymans*, and *X. cucurbita*) were routinely cultured on nutrient agar (NA) or Luria Bertani (LB) agar, while fungal strains (*F. solani*, *F. oxysporum*, *S. cucurbitacearum*, *B. cinerea*, and *E. rostratum*) were maintained on potato dextrose agar (PDA). Plant viruses included Cucumber green mottle mosaic virus (CGMMV), Cucumber mosaic virus (CMV), Potato virus X (PVX), Turnip mosaic virus (TuMV), Pepper mild mottle virus (PMMoV), and Tobacco mosaic virus (TMV), with symptomatic leaf samples provided by Ningbo University Institute of Plant Virology. Prior to experimental use, all pathogens underwent molecular verification to ensure strain correctness.

Bacterial genomic DNA was extracted using the TIANamp Bacteria DNA Kit (Tiangen, China), and fungal DNA was isolated with the SIMGEN Plant DNA Kit (SIMGEN, China). Total RNA was extracted using the TIANamp Virus RNA Kit (Tiangen, China), followed by cDNA synthesis using the TIANscript RT Kit (Tiangen, China). Gel extraction was performed using the SIMGEN Gel Extraction Kit (SIMGEN, China). The *A. citrulli* 16S-23S rRNA intergenic spacer region (ISR) DNA sequence served as served as our diagnostic target, since this locus is widely utilized in bacterial diagnostics due to its sequence variability flanked by conserved ribosomal genes. For sensitivity testing, we used Plasmid-T, a recombinant plasmid containing a 343-bp fragment of the *A. citrulli* 16S-23S rRNA ISR DNA (NCBI:_JQ743876.1). Plasmid concentration was quantified via the Qubit dsDNA BR Assay Kit (Invitrogen, USA), with copy numbers calculated as: copies/μL = [c (ng/μL) × 6.02 × 10^23^ (copies/mol) × 10^-9^ (g/ng)] / [(plasmid length (bp) × 660 (dalton/bp)] (Ding et al., 2024).

All DNA oligonucleotides, including primers, partzymes, synthetic ssDNA target and probes (Table S1) employed in this study, were synthesized by Sangon Biotech (Shanghai, China). For comparative analysis, we employed the previously published fluorescent RPA system using primers Ac-F3/Ac-R2 and probe Ac-P (Wang et al., 2022). Our OAR-MNA biosensor incorporated optimized *A. citrulli*-specific RPA primers AC-SP-2/AC-AP-2 (Yang et al., 2024), with AC-P A/B effector-binding arms designed using Primer Premier 5 software based on RPA amplicon sequences. The specificities of the primers were assessed using NCBI Primer-BLAST ([www.ncbi.nlm.nih.gov/tools/primer-blast/](https://www.ncbi.nlm.nih.gov/tools/primer-blast/)). MNA fluorescence quencher (FQ)-probes were prepared by thermal annealing (95 ℃ followed by gradual cooling to 25 ℃) to ensure proper hairpin formation. An 80-nt synthetic AC-ssDNA target contained complete binding sites for all designed AC-P sequences.

The MIRA DNA Isothermal Rapid Amplification Kit (for RPA assay), MIRA DNA Isothermal Rapid Amplification Fluorescence Kit (for Fluorescent RPA assay) and MIRA RNA Isothermal Rapid Amplification Kit-II (for RT-RPA assay) were from Amp-future Biotech Co., Ltd. (Changzhou, China). The FastStart Essential DNA Probes Master (Roche, Germany) was used for qPCR assays. RNA transcribed was performed using the RiboMAX™ Large Scale RNA Production System (Promega, USA). Fluorescence signal monitoring of all assays (fluorescent RPA, qPCR and OAR-MNA biosensor) was performed on a Bio-Rad CFX96 system, with fluorescent images captured by the Bio-Rad ChemiDoc MP Imaging System. Agarose electrophoresis images were documented using the Bio-Rad XR+ gel imaging system.

**1.2 RPA assay procedures**

Standard RPA reactions were performed according to the manufacture’s instructions using MIRA DNA Isothermal Rapid Amplification Kit. Each reaction mixture contained 29.4 μL of rehydration A buffer, one pellet with recombinase and polymerase, 2 μL of each 10 μM *A. citrulli* forward and reverse RPA primers, 2 μL of DNA template, 2.5 μL B-buffer containing MgOAc, and double-distilled water to a final volume of 50 μL. After vortexing and centrifugation, reactions were incubated at 40 ℃ for 30 min.

For asymmetric RPA, reaction conditions mirrored the standard assay except for reverse primer concentration. We evaluated ssDNA yield using various forward to reverse primer ratios (nM): 10,000:10,000, 10,000:2,000, 10,000:1,000, 10,000:500, 10,000:250, 10,000:125, 10,000:62.5, and 10,000:31.25 using 2,000 copies/μL template DNA, followed by electrophoretic analysis to determine the optimal conditions. To validate robustness, *A. citrulli* DNA dilutions (ranging from 2x10^0^ to 2x10^6^ copies/μL) were tested with the selected primer ratio (10,000 nM:31.25 nM). The amplified products were extracted using DNA Extraction Reagent (Phenol: Chloroform: Isoamylol=25:24:1) and subsequently analyzed on 2% agarose gels.

For RT-RPA, a 50 μL reaction using the MIRA RNA Isothermal Rapid Amplification Kit-II contained: 29.4 μL rehydration A buffer, 2 μL of each RPA primer (10 μM), 2 μL RNA template, 12.1 μL nuclease-free water, one enzyme pellet, and 2.5 μL MgOAc-containing B-buffer.

Fluorescent RPA reaction (50 µL) employed the MIRA DNA Isothermal Rapid Amplification Fluorescence Kit with: 29.4 µL of rehydration A buffer, 11.5 µL of nuclease-free water, 2 µL of each primer (10 μM), 0.6 µL of probe (10 μM), 2 µL of DNA template, 2.5 µL of B-buffer containing MgOAc and one enzyme pellet. Real-time amplification was monitored on a Bio-Rad CFX96 system (40 cycles, 40 s/read).

**1.3 AC-Partzyme screening and ssDNA-based MNAzyme assay optimization**

The initial MNAzyme reaction mixture, with a volume of 20 μL, included 500 nM AC-P A/B, 500 nM MNA FQ-probe, 100 nM AC-ssDNA, and MNAzyme buffer with 100 mM MgCl_2_. The AC-P A/B pairs were initially screened, and the optimal AC-P A/B pair was selected based on fluorescent signals. Subsequent optimizations focused on AC-P A/B concentrations, exploring ranges from 125 nM to 1,000 nM to determine the optimal levels for maximal fluorescence signals. Temperature optimization was conducted over a gradient from 35 ℃ to 60 ℃, in 5 ℃ increments. The optimization of the MNA FQ-probe was evaluated using four concentration gradients: 125 nM, 250 nM, 500 nM, and 1,000 nM. The reaction volume of the MNAzyme was determined using three volume gradients: 20 μL, 30 μL, and 40 μL. The optimal conditions were established by fluorescence and the F/F_0_ ratio.

**1.4 Optimization of the OAR-MNA biosensor**

To achieve optimal performance of the OAR-MNA biosensor, subsequent experiments were conducted, including the reaction temperature, the concentration of Mg^2+^, AC-P A/B, and MNA FQ-probe, the volume ratio of aRPA/MNAzyme, and the aRPA primer concentration ratio. Firstly, the temperature was fine-tuned from 37 ℃ to 42 ℃ in 1 ℃ increments to select the optimal temperature. The optimal concentration of Mg^2+^ was determined by testing six concentration gradients: 0 nM, 20 nM, 40 nM, 80 nM, 160 nM, and 320 nM. The volume ratio of aRPA and MNAzyme varied at 10 μL:40 μL, 20 μL:40 μL, and 40 μL:40 μL. The ratios of forward to reverse primers in nM were set as follows: 10,000:250, 10,000:125, 10,000:62.5, and 10,000:31.25. The optimization of AC-P A/B and MNA FQ-probe was evaluated by using four concentration gradients: 125 nM, 250 nM, 500 nM, and 1,000 nM.

**1.5 Molecular mechanism analysis of OAR-MNA specificity and fluorescent RPA cross-reactivity**

To elucidate the molecular basis for OAR-MNA's species discrimination capability versus fluorescent RPA's cross-reactivity, we performed targeted sequencing of amplification products. Target RPA-amplified bands (dsDNA) from Ac-F3/Ac-R2 primer amplification of *Acidovorax citrulli* (Ac) or *A. avenae* subsp. *avenae* (Aa) were excised and gel-purified and sequenced via paired-end assembly. Subsequently, target aRPA-amplified bands (ssDNA) were similarly processed and subjected to Sanger sequencing.

**1.6 Performance of OAR-MNA biosensor on real seed samples**

To verify the applicability of the OAR-MNA biosensor, twenty-two watermelon seed samples were tested for *A. citrulli* contamination. These seeds underwent pretreatment described as follows: the watermelon seeds were lightly crushed using a micro-grinder and completely immersed in PBS solution to facilitate bacterial release. The soaking solution was centrifuged at 1,000 rpm to remove any precipitate, and the supernatant was re-centrifuged at 10,000 rpm; the pellet was suspended in sterile water. DNA was extracted using the TIANamp Bacteria DNA Kit (Tiangen, China). The obtained DNA was used as a template for both the OAR-MNA biosensor and qPCR assay to test the accuracy of the OAR-MNA biosensors.

**1.7 Performance of OAR-MNA biosensor on spiked watermelon homogenate samples**

Healthy watermelons without spots on the surface were selected, washed with 75% ethanol, and then rinsed with deionized water to remove surface residues. Watermelons, including rind and pulp, were cut into 30-g portions using a sterilized scalpel. The watermelon pieces were slightly crushed with a pulverizer to obtain watermelon homogenate samples, which were confirmed to be negative for *A. citrulli* using the qPCR method.

The *A. citrulli* strain was cultured, and 10-fold serial dilutions were performed using PBS to obtain suspensions of different concentrations. For each experiment, 8 mL of watermelon homogenate was mixed with 2 mL of bacterial solution at varying concentrations. Subsequently, 2 mL of the mixture was sampled for DNA extraction. The extracted DNA was then analyzed by both OAR-MNA biosensor and qPCR assay.

**1.8 qPCR toward BFB detection**

The primers and probe used in qPCR assay were also listed in Table S1. In the qPCR assay, the reagents were added following the manufacturer’s instructions. A qPCR mixture contained 12.5 μL qPCR Premix, 1 μL of each 10 μM AC-FP and AC-RP, 0.5 μL of 10 μM AC-qP, 1 μL template, and double-distilled water to a final volume of 25 μL. The reaction conditions consisted of an initial denaturation step at 95 ℃ for 10 min, followed by 40 cycles of 15 s at 94 ℃ and 60 s at 58 ℃. Fluorescence was recorded at the end of every cycle. The fluorescent photographs were imaged by the Bio-Rad ChemiDoc MP Imaging System with its built-in UV channel.

**1.9 Application of OAR-MNA in Cucumber green mottle mosaic virus (CGMMV) detection**

To validate the broad applicability of the OAR-MNA assay across pathogen types, we adapted the system for detection of Cucumber green mottle mosaic virus (CGMMV), a globally significant RNA virus causing substantial economic losses in cucurbit crops. By employing the MIRA RNA Isothermal Rapid Amplification Kit-II containing integrated reverse transcriptase, we established RNA target detection capability without protocol modifications beyond kit substitution.

The CGMMV coat protein region (nt 5684-6337; GenBank GCA_000849225.1) served as the diagnostic target. Initial amplification with primers CGMMV-SP1/ CGMMV-AP1 (Table S1) produced an amplicon that was cloned into the vector and sequence-validated by Sangon Biotech. For *in vitro* transcription templates, amplification with T7 promoter-tagged primers CGMMV-T7-SP1/CGMMV-AP1 was performed, with amplicons purified via gel electrophoresis before transcription using the RiboMAX™ Large Scale RNA Production System (Promega). Purified transcripts were quantified for downstream use.

Following design of CGMMV-specific RPA primers and partzymes (Table S1, validated by NCBI Primer-BLAST), optimal primers were screened in 50 μL RT-RPA reactions. Subsequently, optimal partzymes were screened in OAR-MNA assays combining 10 μL RT-RPA components with 40 μL MNAzyme mixture (250 nM each CGMMV-P A/B, 500 nM FQ-probe).

Specificity was assessed using total RNA extracted from 100 mg symptomatic leaf tissues. For field validation, 13 watermelon seed samples were analyzed using parallel qPCR and OAR-MNA assays. Total RNA extracted from 100 mg seed powder was directly used for OAR-MNA, while an aliquot was reverse-transcribed for qPCR.

1. **Supplementary Figures and Tables**


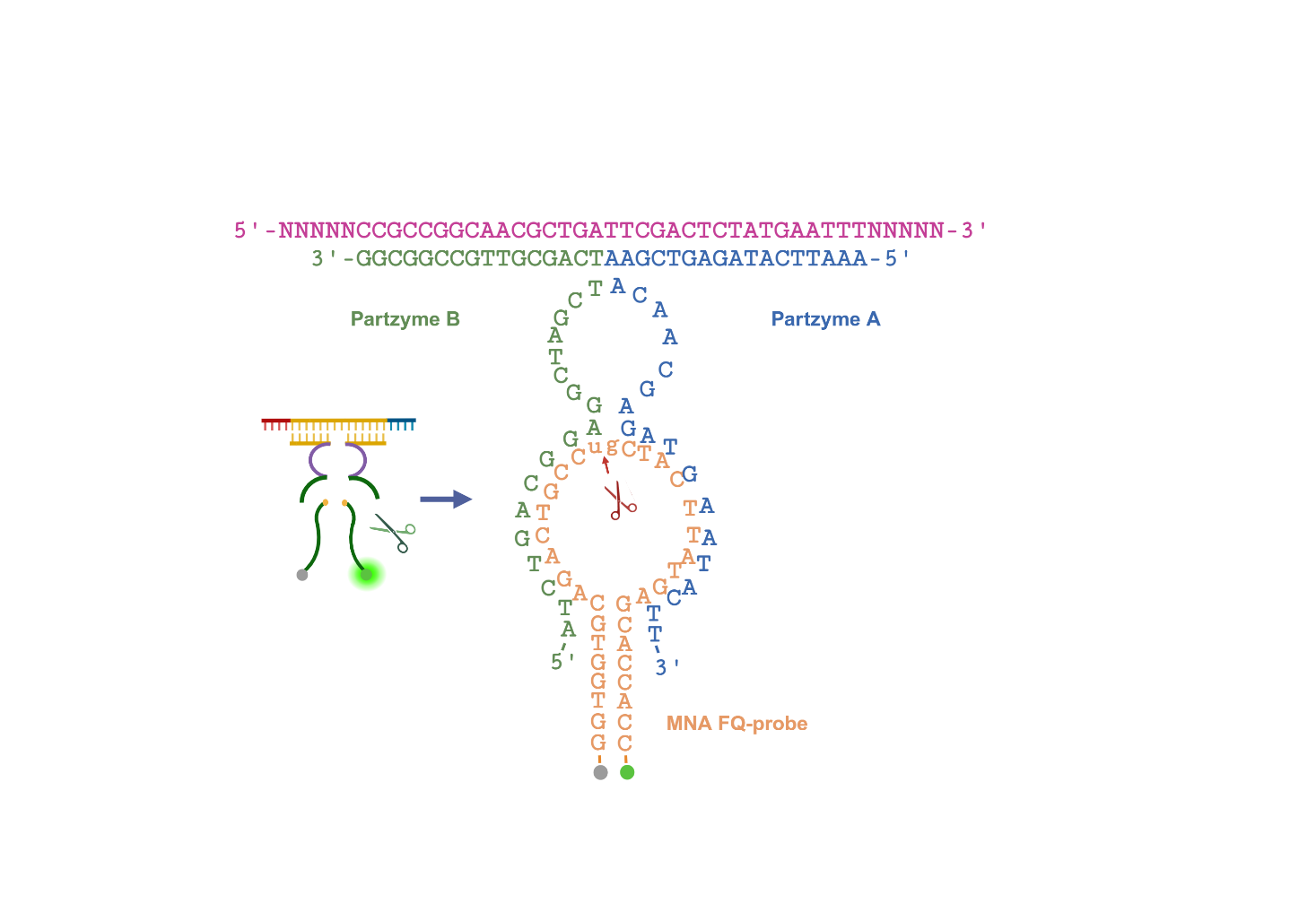


**Figure S1. Schematic illustration of the specific designs of the base sequences of MNA FQ-probe, partzyme A/B and target ssDNA for triggering the cleavage reaction.**


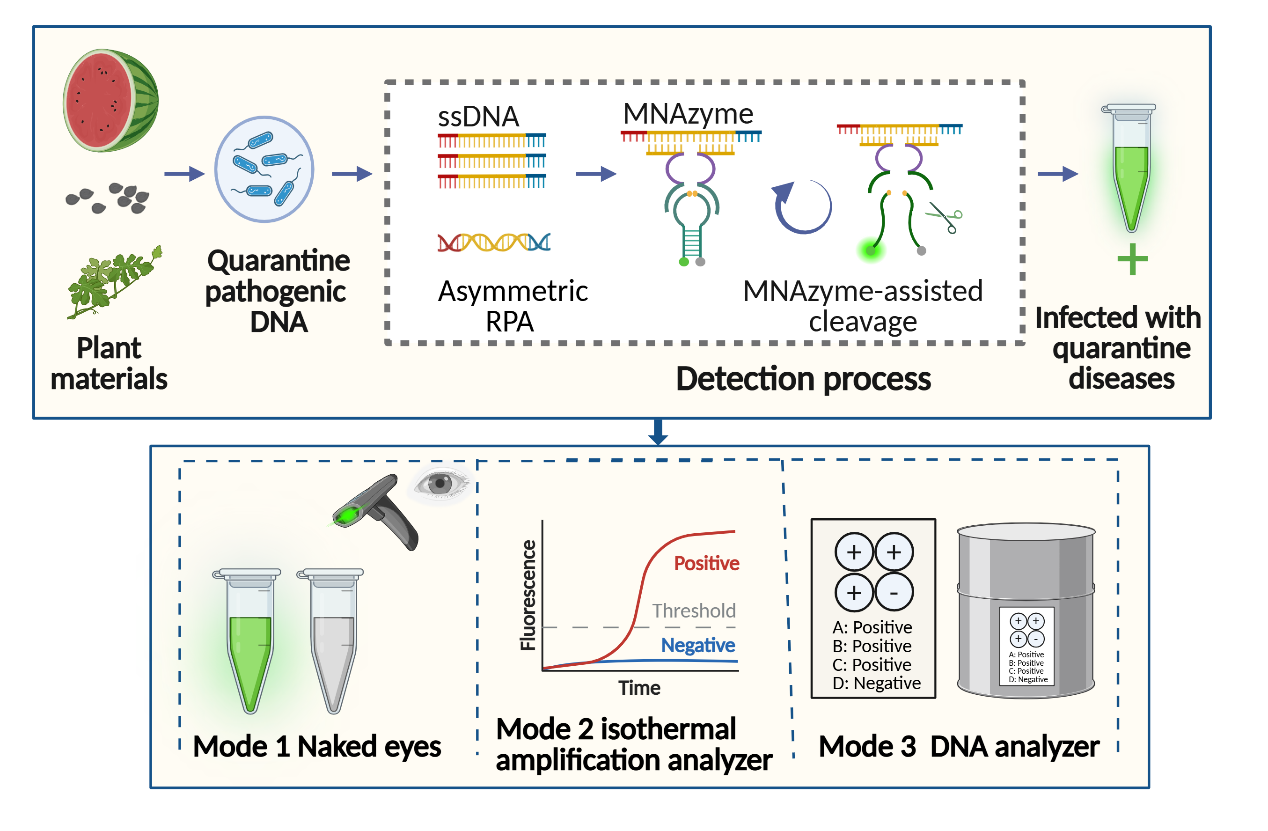


**Figure S2. The result output mode of the OAR-MNA biosensor.** Mode 1: Visual detection under UV lamp; Mode 2: Analysis via real-time PCR; Mode 3; Detected through a pocket-size DNA analyzer.


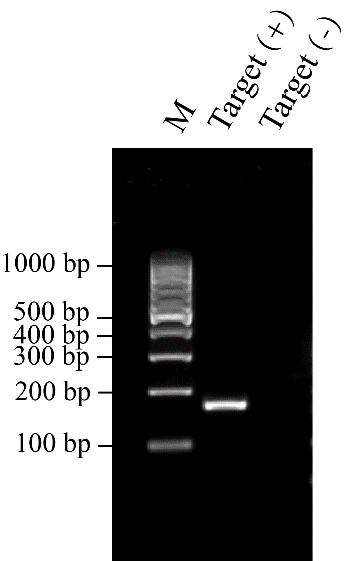


**Figure S3. Electrophoresis of RPA amplicon using *Acidovorax citrulli* specific RPA primers (AC-SP-2/AC-AP-2) with or without target.**


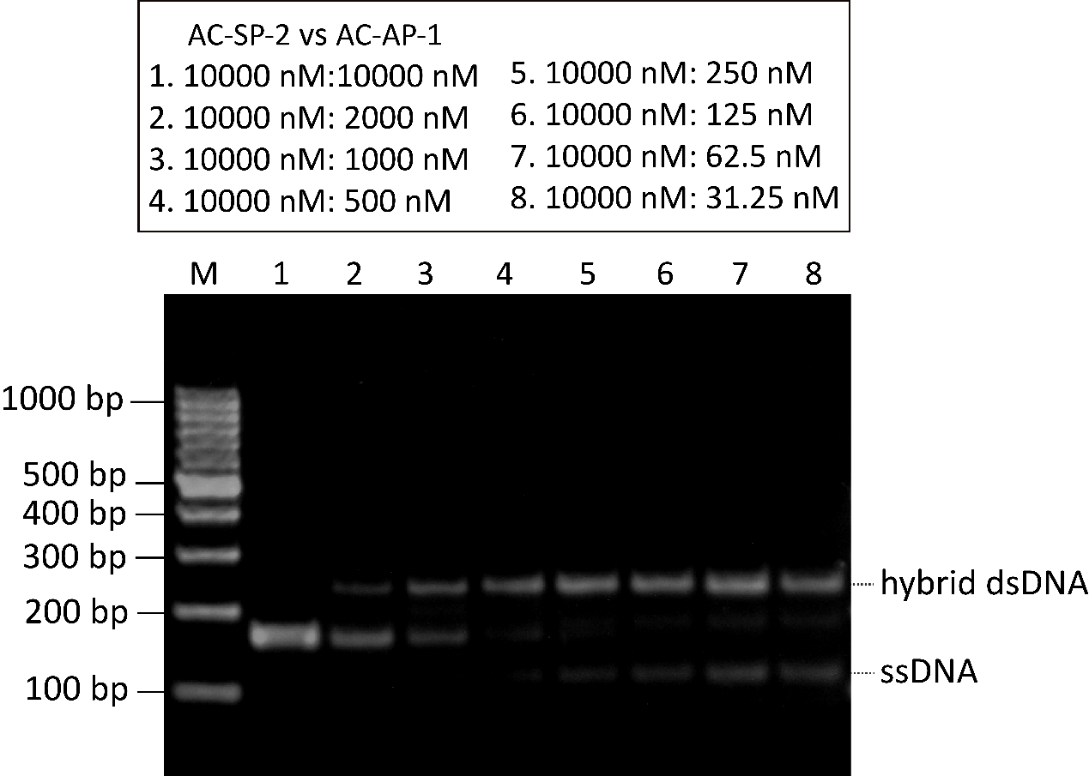


**Figure S4. Primer ratio optimization for aRPA.** Amplification visualized on agarose gel, with reverse to forward primer ratios ranging from 10,000 nM:10,000 nM (1:1) to 10,000 nM:31.25 nM (320:1). M: 100-1,000 bp marker; 1: 10,000:10,000 nM; 2: 10,000:2,000 nM; 3: 10,000:1,000 nM; 4: 10,000:500 nM; 5: 10,000:250 nM; 6: 10,000:125 nM; 7: 10,000:62.5 nM; 8: 10,000:31.25 nM.


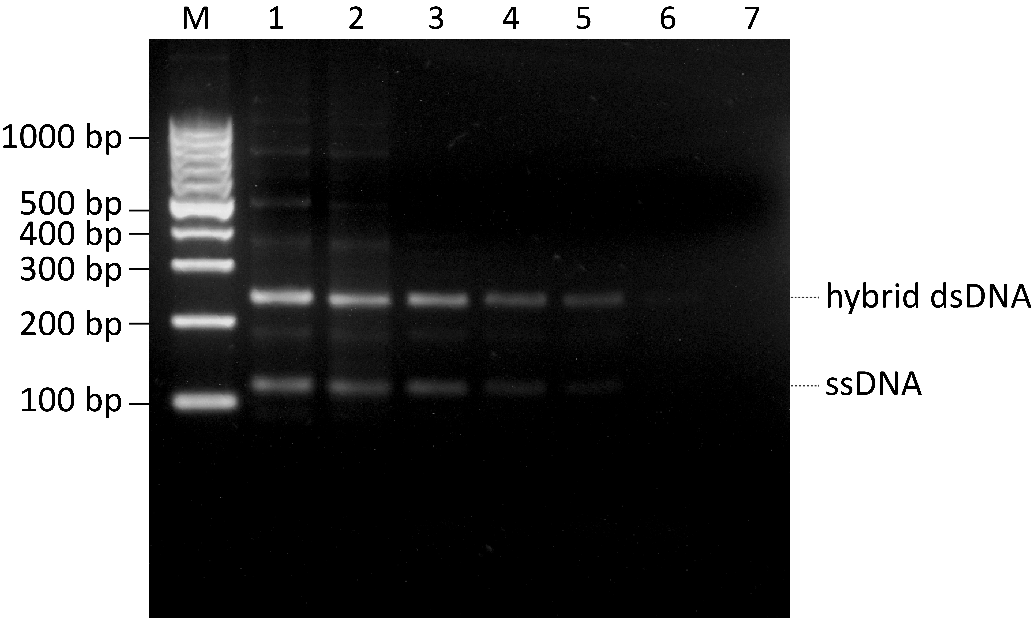


**Figure S5. Dynamic range assessment of ssDNA yield by testing template concentrations across a wide range (2-2×10⁶ copies/μL) using a primer ratio of 320 (10,000 nM) :1 (31.25 nM).** M: 100-1,000 bp marker; 1: 2x10^6^ copies/μL; 2: 2x10^5^ copies/μL; 3: 2x10^4^ copies/μL; 4: 2x10^3^ copies/μL; 5: 2x10^2^ copies/μL; 6: 2x10^1^ copies/μL; 7: 2x10^0^ copies/μL.

**Figs. S6-S8: AC-P A/B screening and ssDNA-based MNAzyme reaction parameter optimization**

To enhance the analytical performance of the OAR-MNA biosensor for BFB detection, key parameters of the ssDNA-based MNAzyme reaction were tuned using fluorescence intensity and F/F₀ as metrics (Figs. S6, S7). Among AC-P A/B variants 1-3, AC-P A3/B3 exhibited the highest fluorescence intensity and F/F₀ (Figs. S6A, S7A) and was selected for further tests. Fluorescence intensity and F/F₀ increased with AC-P A3/B3 concentrations from 125 to 250 nM but decreased beyond 250 nM (Figs. S6B, S7B), setting 250 nM as optimal. The reaction performed best at 40 ℃ (Figs. S6C, S7C). While fluorescence intensity rose with probe concentration, F/F₀ peaked at 500 nM (Figs. S6D, S7D), which was chosen as the optimal probe concentration.

For MNAzyme reaction volume, fluorescence intensity and F/F₀ increased from 20 to 40 µL (Figs. S6E, S7E). To achieve one-pot detection of the OAR-MNA biosensor, the stability of the MNAzyme components on the tube lid was evaluated. It was found that 20-40 µL of MNAzyme components adhered reliably to the tube lid, while larger volumes (50-60 µL) did not (Fig. S8). Therefore, 40 µL was selected to ensure reliable performance.


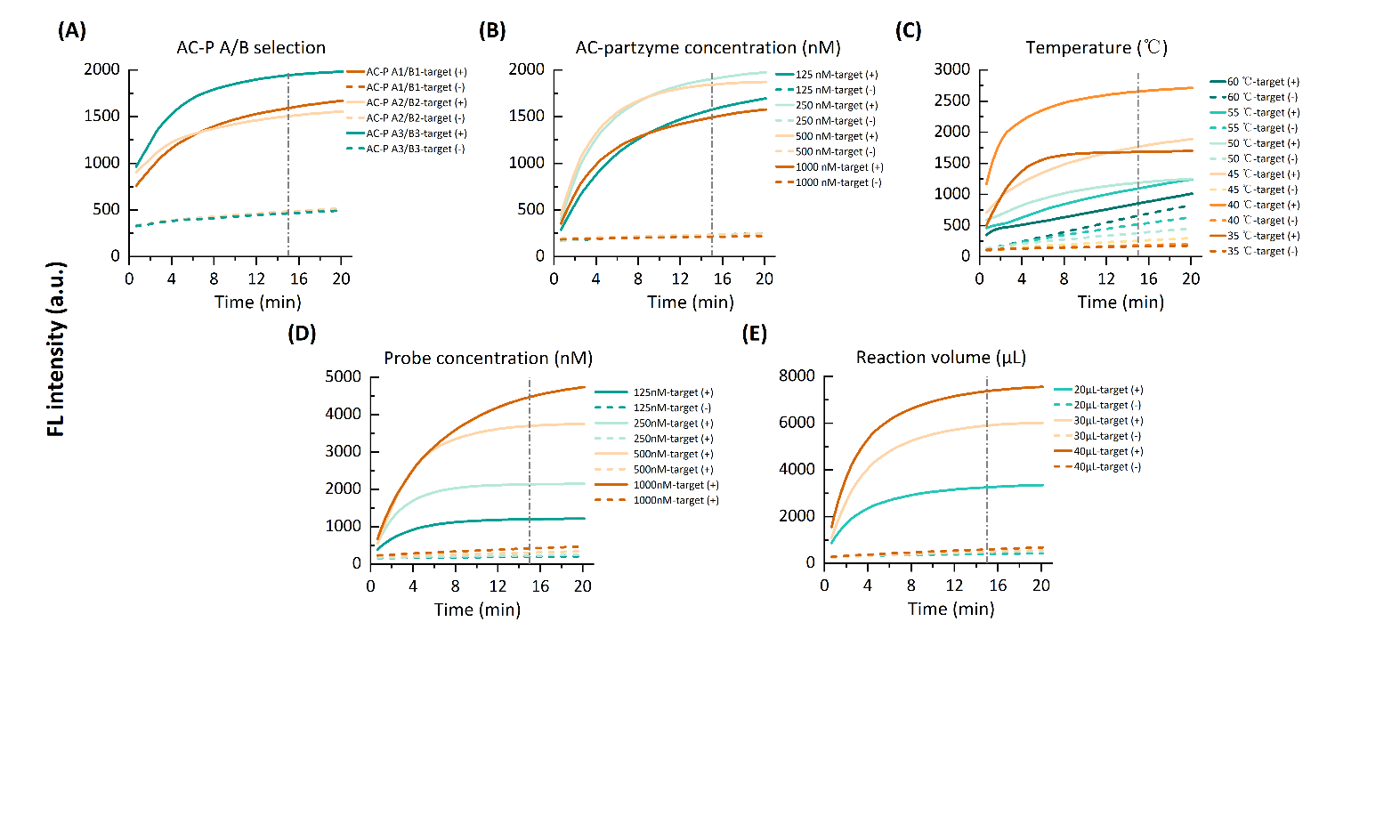


**Figure S6. Real-time curves of the ssDNA-based MNAzyme with different reaction conditions.** (A) Selection of AC-P A/B pair, (B) AC-P A/B concentration, (C) Optimal working temperature, (D) Probe concentration, (E) Reaction volume. A total of 20 minutes of fluorescence curves were recorded, with 15 minutes being the selected time point in Figure S7.


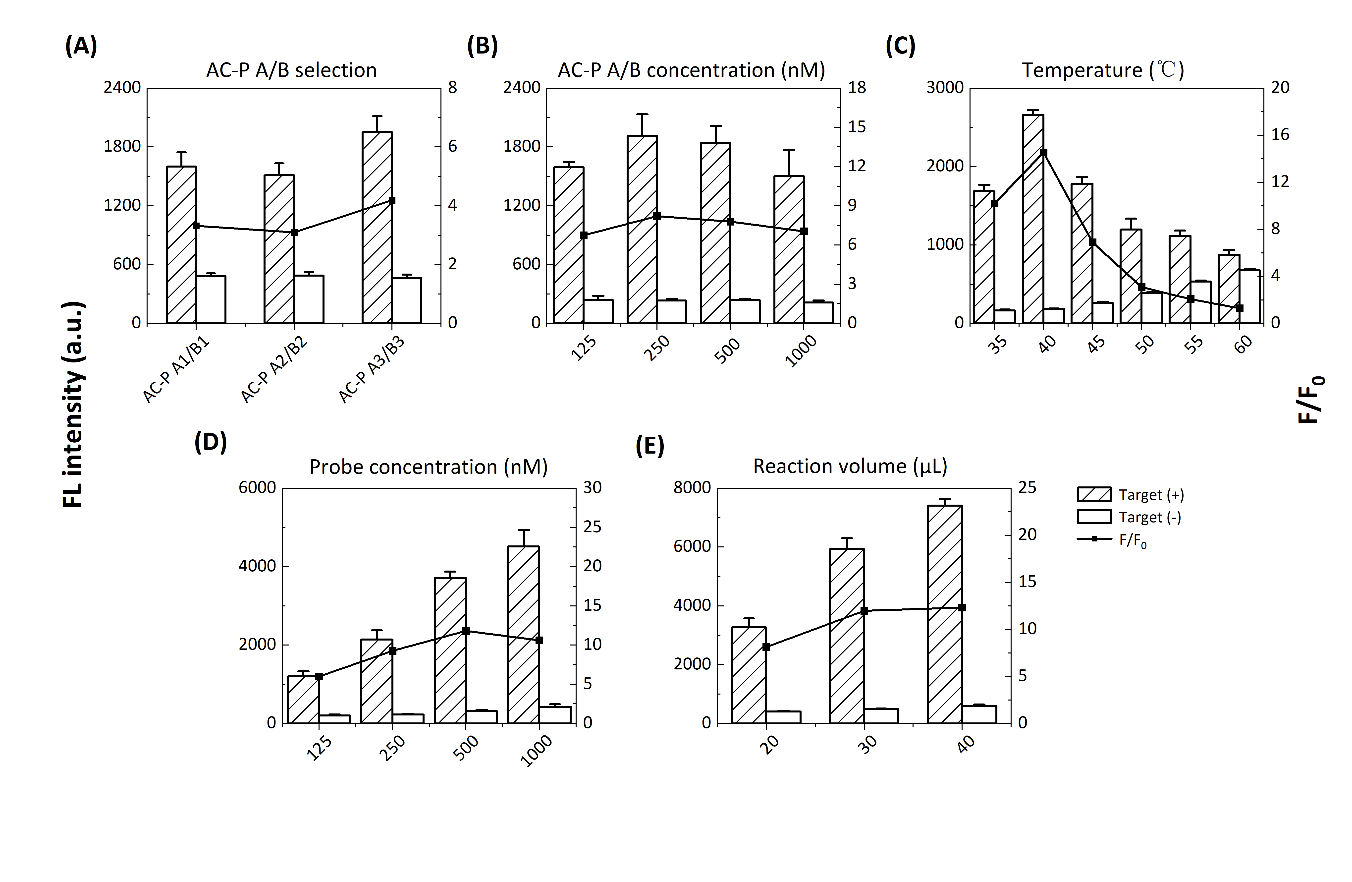


**Figure S7. Optimizing ssDNA-based MNAzyme reaction conditions.** (A) Selection of AC-P A/B pair, (B) AC-P A/B concentration, (C) Optimal working temperature, (D) Probe concentration, (E) Reaction volume. Error bars present means ± standard deviation from triplicate experiments. (F: FL intensity of the corresponding conditions in the presence of target; F_0_: blank FL intensity of the corresponding conditions without target).


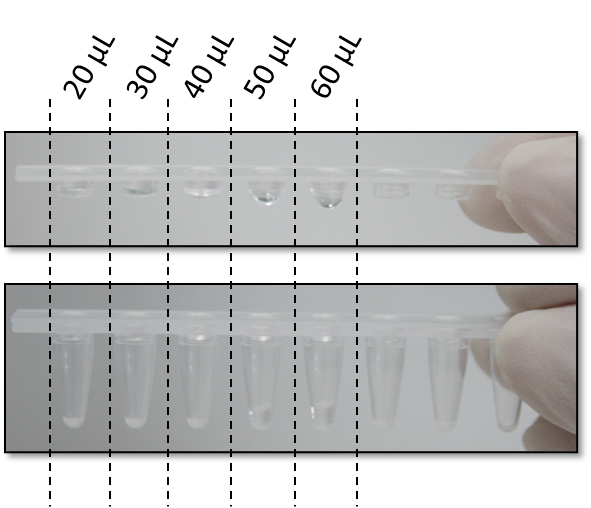


Figure S8. Stability of MNAzyme reagents on 8-strip PCR tube lid. Droplet of the MNAzyme reagent at 20-40 μL was maintained well on the lid of the tube and did not fall off when the lid attached to the tube. In contrast, 50 and 60 μL of MNAzyme reagent formed unstable droplets on the lid, which tended to fall off when the lid was attached to the tube.


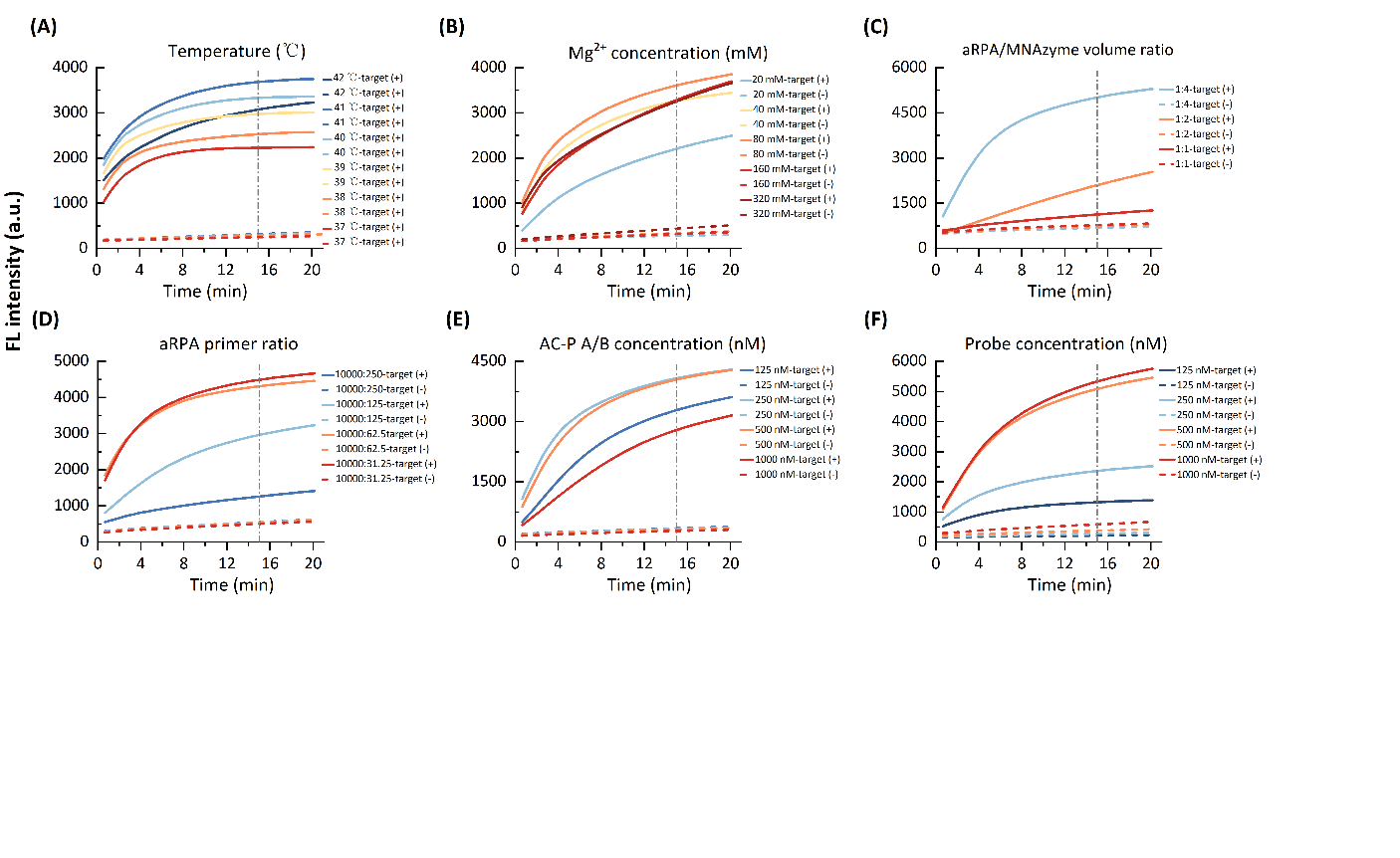


**Figure S9. Real-time curves of the OAR-MNA biosensor with fine-tuned key parameters.** (A) Selection of optimal working temperature, (B) Mg^2+^ concentration, (C) Volume ratio of aRPA and MNAzyme, (D) aRPA primer ratio, (E) Partzyme concentration and (F) Probe concentration. A total of 20 minutes of fluorescence curves were recorded, with 15 minutes being the selected time point in Figure 3.


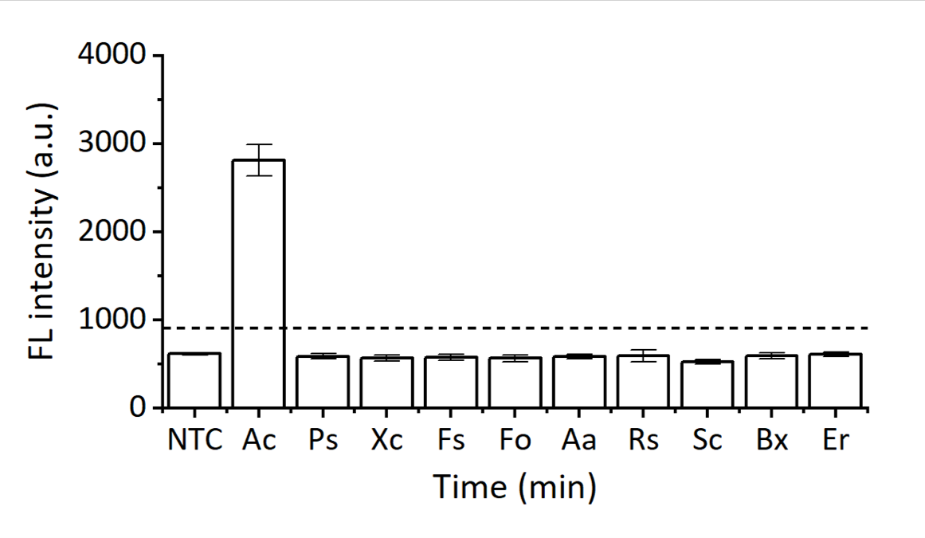


**Figure S10. OAR-MNA biosensor specificity for BFB detection.** Bar charts of the OAR-MNA endpoint assay. Error bars represent the means ± standard deviations from triplicate independent experiments, with NTC (non-template control reaction) as control. The horizontal dashed line indicates the threshold fluorescence intensity (NTC + 3SD). Ac: *Acidovorax citrulli*; Ps: *Pseudomonas syringae*; Xc: *Xanthomonas cucurbita*; Fs: *Fusarium solani*; Fo: *Fusarium oxysporum*; Aa: *Acidovorax avenae subsp. avenae*; Rs: *Ralstonia solanacearum*; Sc: *Stagonosporopsis cucurbitacearum*; Bc: *Botrytis cinerea*; Er: *Exserohilum rostratum*.


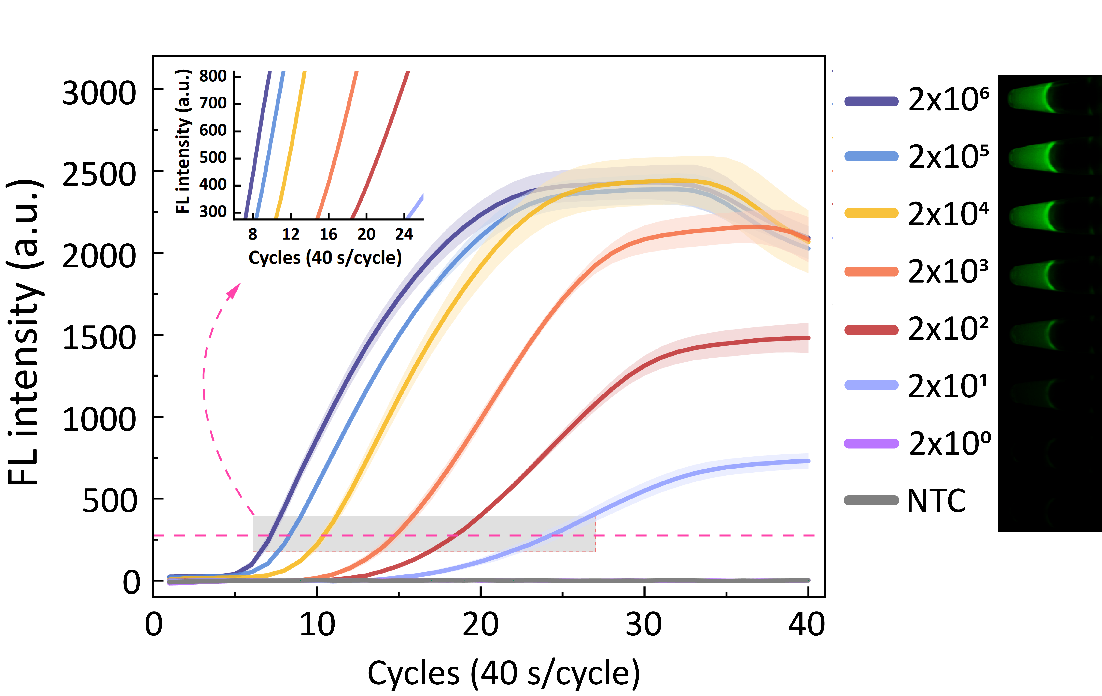


**Figure S11. Sensitivity detection of *Acidovorax* *citrulli* via fluorescent RPA assay.** Real-time detection curves and fluorescence images at Plasmid-T DNA concentrations from 2-2×10⁶ copies/μL. Error bars represent the means ± standard deviations from triplicate independent experiments, with NTC (non-template control reaction) as control.


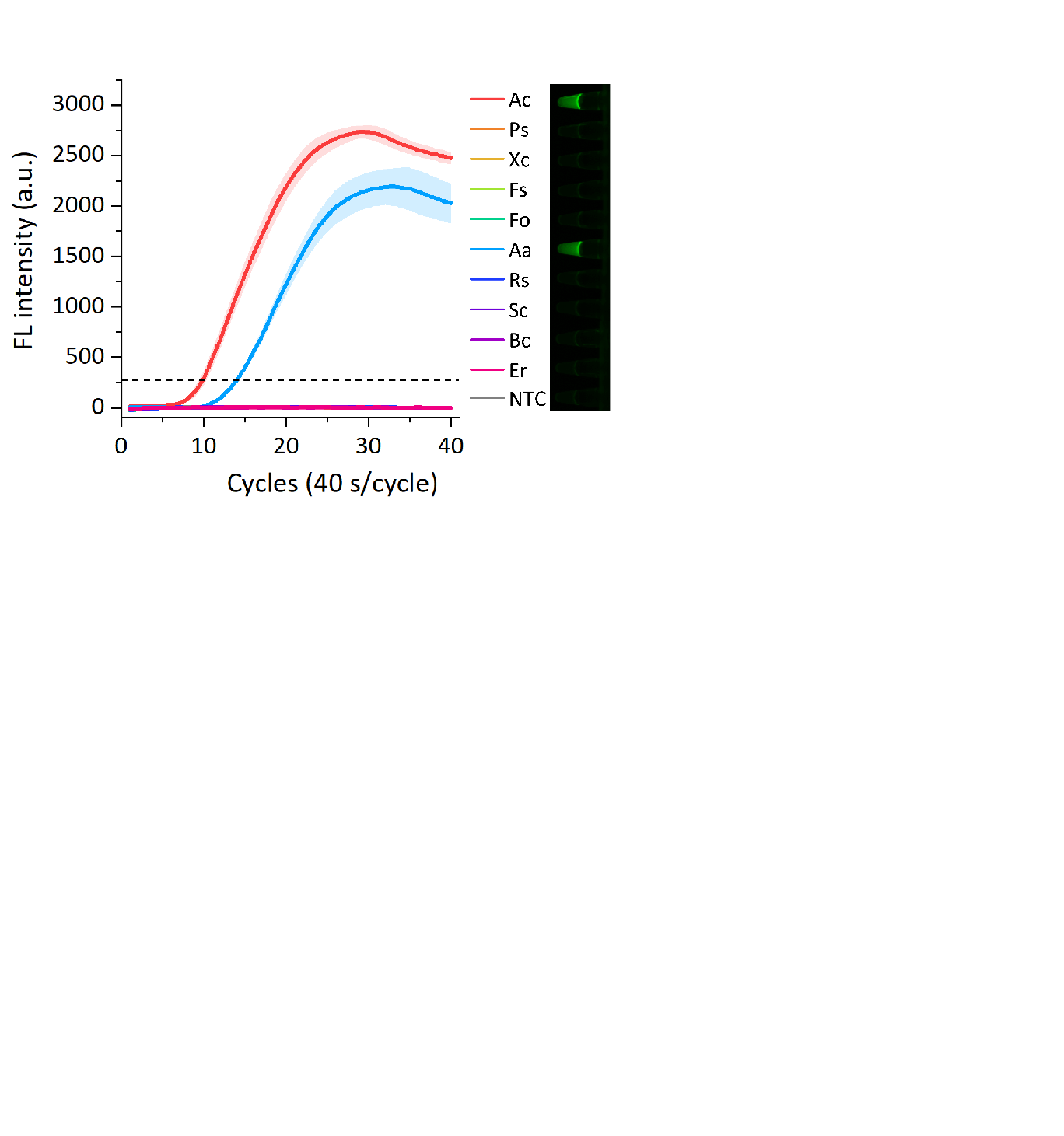


**Figure S12. Specificity assessment of *Acidovorax* *citrulli* via fluorescent RPA assay.** Real-time detection curves and fluorescence images with ten related pathogens. Error bars represent the means ± standard deviations from triplicate independent experiments, with NTC (non-template control) as control. Ac: *Acidovorax citrulli*; Ps: *Pseudomonas syringae*; Xc: *Xanthomonas cucurbita*; Fs: *Fusarium solani*; Fo: *Fusarium oxysporum*; Aa: *Acidovorax avenae subsp. avenae*; Rs: *Ralstonia solanacearum*; Sc: *Stagonosporopsis cucurbitacearum*; Bc: *Botrytis cinerea*; Er: *Exserohilum rostratum*.

**Figs. S13-S19: Investigating fluorescent RPA cross-reactivity and OAR-MNA specificity.**

Systematic electrophoretic and sequencing analyses revealed the molecular basis for fluorescent RPA's cross-reactivity and OAR-MNA's high specificity. Gel electrophoresis (Fig. S13) confirmed amplification of both *A. citrulli* and *A. avenae* subsp. *avenae* using Ac-F3/Ac-R2 primer set, yielding expected ~160 bp products. Target bands (Fig. S13 dashed regions) were gel-purified and sequenced via paired-end assembly. Results revealed both species containing intact RPA probe binding sites (Fig. S14), explaining fluorescent RPA cross-reactivity.

Since MNAzyme activation requires assembly and cleavage in the presence of target ssDNA, sequencing of excised bands (Fig. S15, dashed regions) was performed. Results showed sequencing using SP-2X yielded no signal, confirming ssDNA structure (Fig. S16), while sequencing using AP-2X revealed critical differences: *A. citrulli* aRPA contained intact MNAzyme targets (AC-P A3/B3), whereas *A. avenae* subsp. *avenae* showed two nucleotide substitutions (Fig. S17). Extensive validation across 36 additional *A. avenae* subsp. *avenae* replicates confirmed consistent adenine (A) substitutions (Fig. S18).

Functional validation using synthetic AC-ssDNA1 and AA-ssDNA1 (truncated sequences corresponding to Fig. S17 white boxes) confirmed that nucleotide substitutions impair MNAzyme cleavage: even at high template concentration (150 nM), only AC-ssDNA1 activated MNAzyme-mediated FQ-probe cleavage (Fig. S19), while substitutions in AA-ssDNA1 prevented stable hybridization and MNAzyme activation, establishing OAR-MNA's absolute specificity.

We propose two plausible mechanisms for the observed adenine substitutions in *A. avenae* subsp. *avenae*: (1) imperfect primer-template complementarity (Fig. S18) predisposing to polymerase errors during amplification, and (2) ssDNA's inherent structural plasticity (versus rigid dsDNA) promoting hairpin formation and tertiary structures that spatially approximate distal nucleotides, altering polymerase fidelity during amplification. These hypotheses offer testable mechanistic frameworks for future investigation of amplification-induced mutation biases.


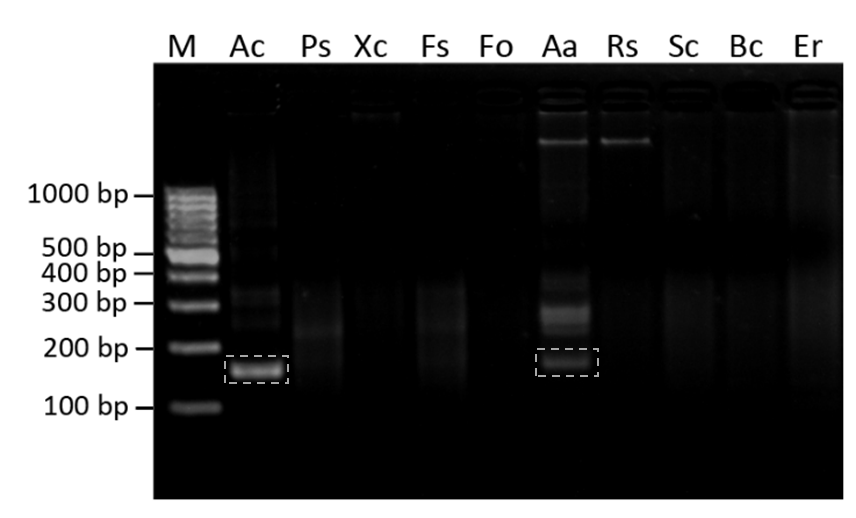


**Figure S13. Gel electrophoresis of RPA amplicons among ten related pathogen species using Ac-F3/Ac-R2 primer set.** Genomic DNAs inputs were adjusted to nearly 2x10^3^ copies/μL. Ac: *Acidovorax citrulli*; Ps: *Pseudomonas syringae*; Xc: *Xanthomonas cucurbita*; Fs: *Fusarium solani*; Fo: *Fusarium oxysporum*; Aa: *Acidovorax avenae subsp. avenae*; Rs: *Ralstonia solanacearum*; Sc: *Stagonosporopsis cucurbitacearum*; Bc: *Botrytis cinerea*; Er: *Exserohilum rostratum*. Dashed boxes indicate bands excised for gel purification and paired-end sequencing.


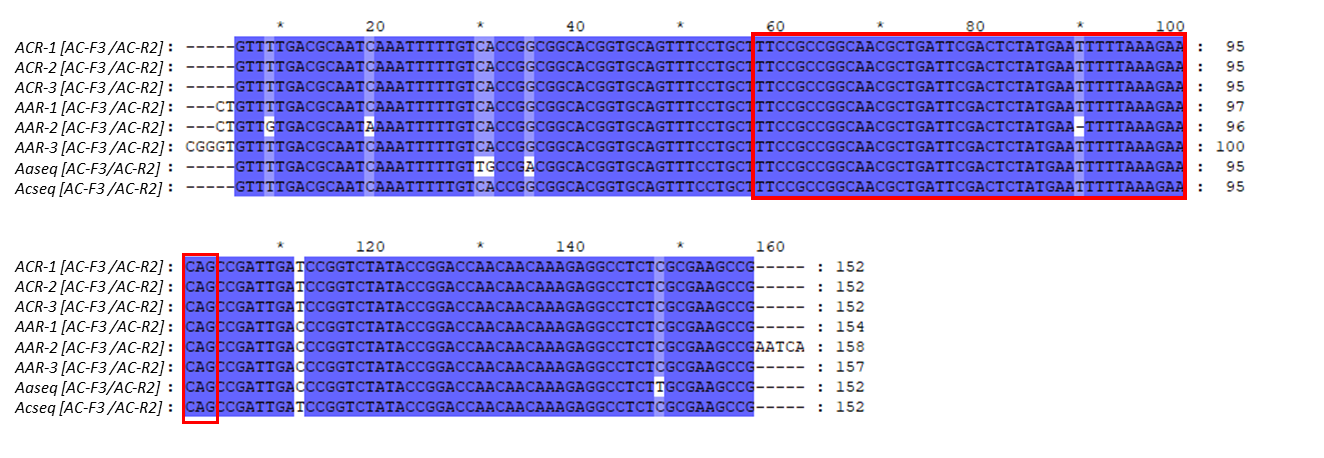


**Figure S14. Sequence alignment of Ac-F3/Ac-R2 RPA amplicons.** Multiple alignment against reference sequences. Aaseq: *Acidovorax avenae* subsp. *avenae* ISR (NCBI: JQ743875.1, positions corresponding to Ac-F3/Ac-R2 amplicon); Acseq: *Acidovorax citrulli* ISR (NCBI: JQ743876.1, positions corresponding to Ac-F3/Ac-R2 amplicon). ACR: *A. citrulli* RPA product; AAR: *A. avenae* subsp. *avenae* RPA product. Sequences derived from paired-end sequencing with assembly using 19-nt primers (F3-X/R2-X). Red box: RPA fluorescent probe target site. Template source: Fig. S13 dashed boxes.


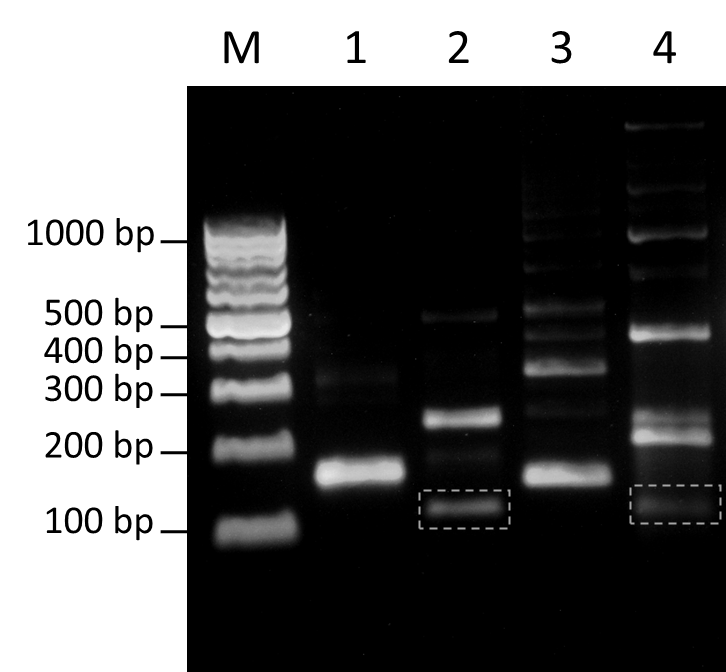


**Figure S15. Electrophoretic comparison of RPA and aRPA products.** Amplification of *Acidovorax avenae* subsp. *avenae* (Aa) and *Acidovorax citrulli* (Ac) using AC-SP-2/AC-AP-2 primer set. Aa and Ac template concentrations standardized to 2×10⁴ copies/μL to enable Aa ssDNA detection. Lane 1: Ac-RPA product; Lane 2: Ac aRPA product; Lane 3: Aa RPA product; Lane 4. Aa aRPA product. Dashed boxes indicate bands excised for gel purification and paired-end sequencing.


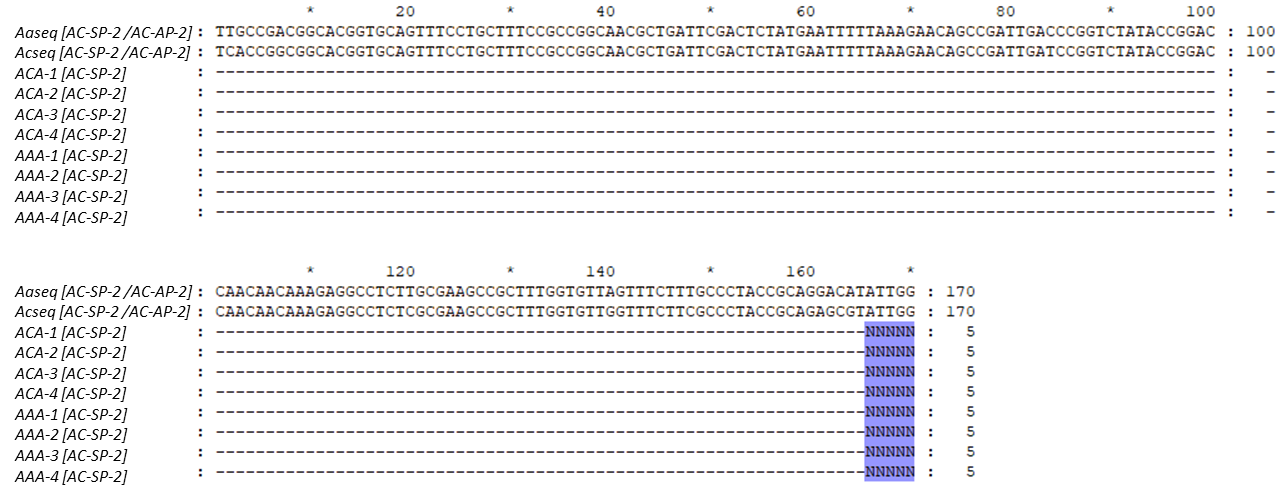


**Figure S16. Sense-strand sequence alignment of aRPA amplicons.** Multiple alignment of *Acidovorax citrulli* (Ac) and *Acidovorax avenae* subsp. *avenae* (Aa) aRPA products against reference sequences. Aaseq: *Acidovorax avenae* subsp. *avenae* ISR (NCBI: JQ743875.1, positions corresponding to AC-SP-2/AC-AP-2 amplicon); Acseq: *Acidovorax citrulli* ISR (NCBI: JQ743876.1, positions corresponding to AC-SP-2/AC-AP-2 amplicon). Sequencing performed with SP-2X primer (terminal 19-nt of AC-SP-2). ACA: aRPA (Ac); AAA: aRPA (Aa).Template source: dashed-box bands in Figure S15.


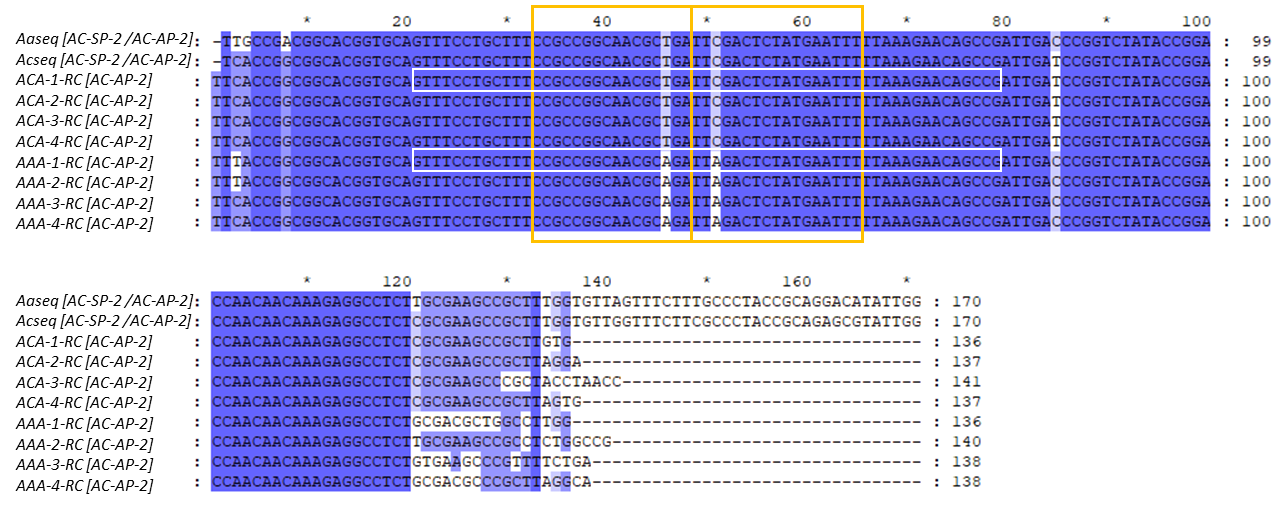


**Figure S17. Antisense-strand sequence alignment highlighting MNAzyme target sites.** Comparison of *Acidovorax citrulli* (Ac) and *Acidovorax avenae* subsp. *avenae* (Aa) aRPA products against reference sequences. Aaseq: *Acidovorax avenae* subsp. *avenae* ISR (NCBI: JQ743875.1, positions corresponding to AC-SP-2/AC-AP-2 amplicon); Acseq: *Acidovorax citrulli* ISR (NCBI: JQ743876.1, positions corresponding to AC-SP-2/AC-AP-2 amplicon). Sequencing performed with AP-2X primer (terminal 19-nt of AC-AP-2). ACA-RC: antisense strand of sequenced Ac aRPA; AAA-RC: antisense strand of sequenced Aa aRPA. Template source: dashed-box bands in Figure S15. Yellow boxes: MNAzyme binding regions. White boxes: Synthetic ssDNA regions (AC-ssDNA1/AA-ssDNA1 derived from truncated ACA-1-RC/AAA-1-RC).


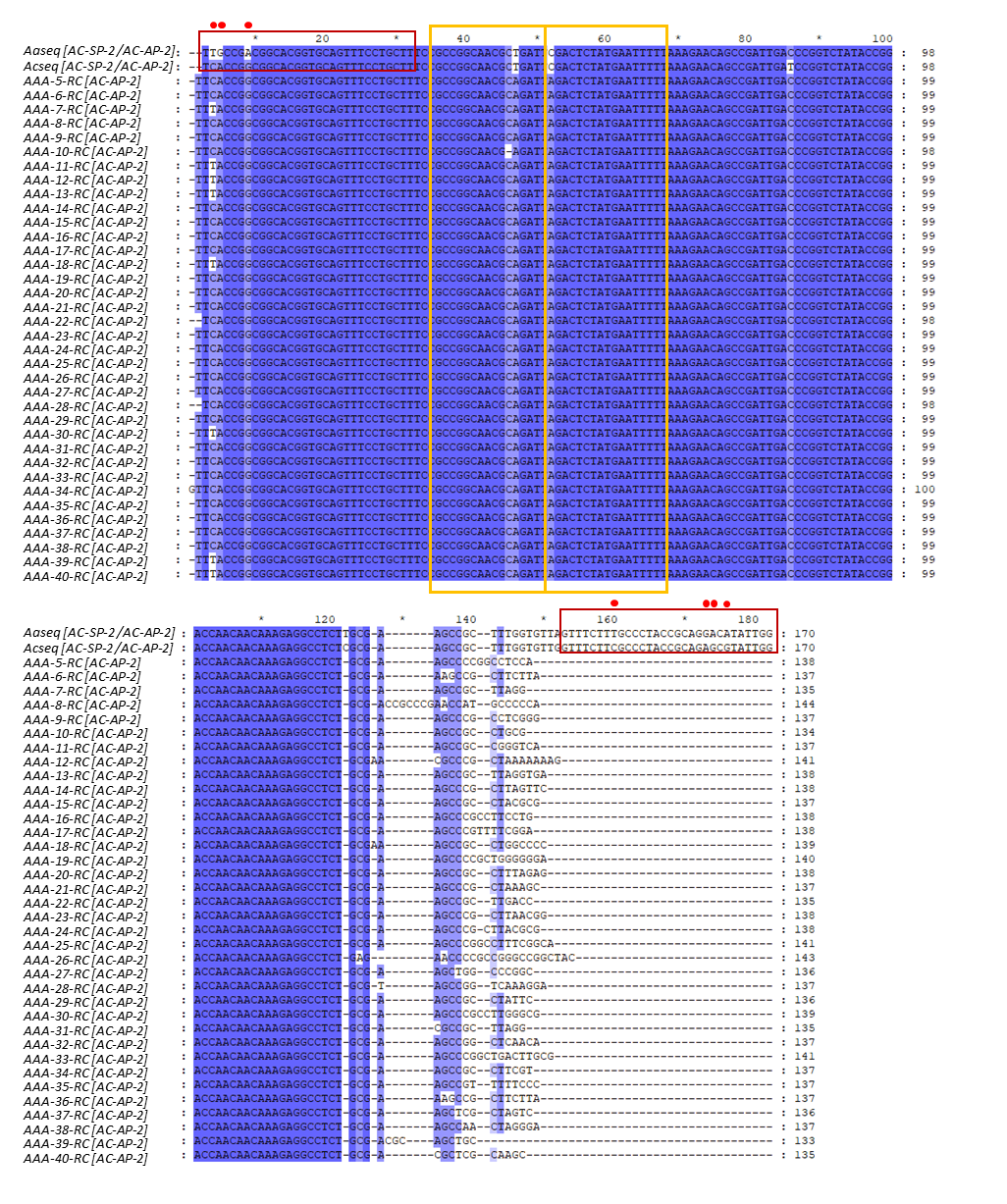


**Figure S18. Additional antisense-strand sequence alignment of *Acidovorax avenae* subsp. *avenae* aRPA amplicons.** Alignment against reference sequences (as in Fig. S17). AAA-RC: antisense strand of sequenced Aa aRPA. Template source: dashed-box bands in Figure S16, Lane 4. Yellow boxes: MNAzyme binding regions. Red boxes: RPA primer binding regions. Red dots: nucleotide differences in primer binding regions.


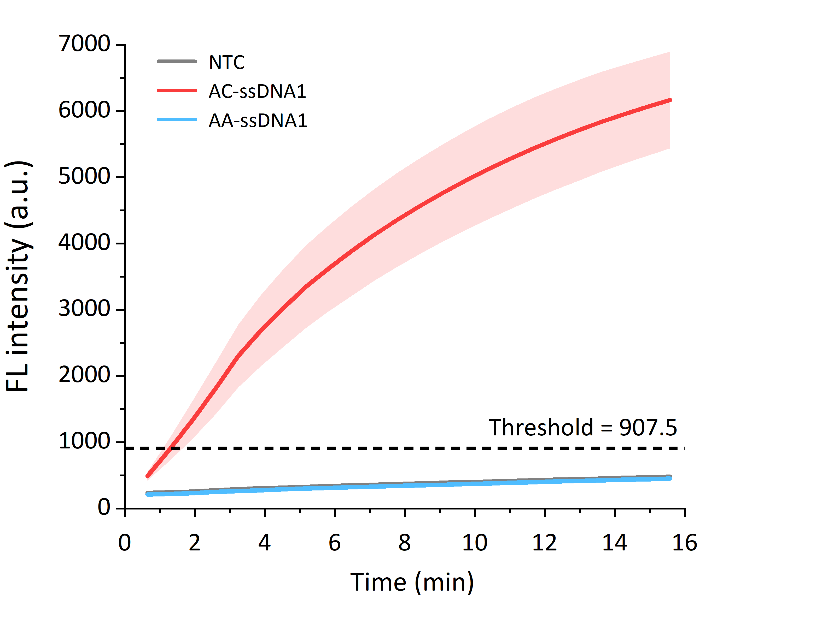


**Figure S19. MNAzyme activation kinetics with synthetic ssDNA.** AC-ssDNA1 and AA-ssDNA1 sequences derived from Fig. S17 white boxes. Template concentration: 150 nM. Fluorescence monitored during MNAzyme cleavage. NTC represents non-template control. The horizontal dashed line indicates the threshold fluorescence intensity.


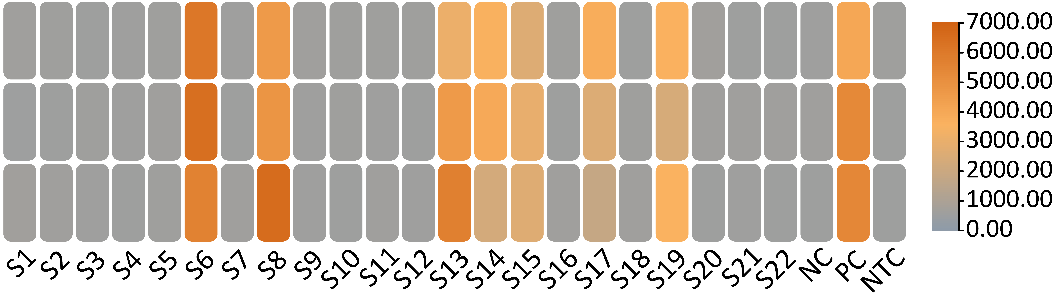


**Figure S20. Heatmap of endpoint fluorescent signals of 22 watermelon seeds detected by the OAR-MNA biosensor.** The color scale represents the intensity of the endpoint fluorescent signals, ranging from low (grey) to high (orange).


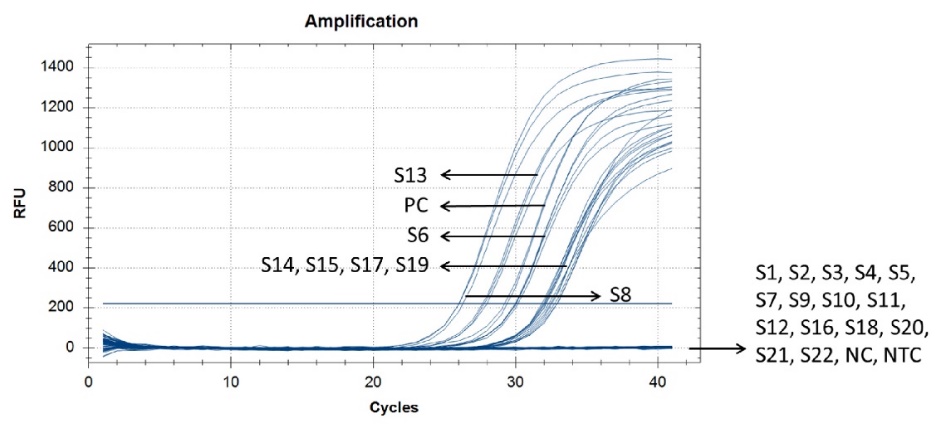


**Figure S21. Real-time curves of the qPCR results for 22 watermelon seeds.** The figure displays the real-time curves obtained from the qPCR analysis of 22 watermelon seed field samples. NC, negative control; PC, positive control; NTC, non-template control. All samples were tested three times.

**Fig. S22-S26: Establishment of OAR-MNA system for CGMMV Detection**

The OAR-MNA system was successfully adapted for CGMMV detection through systematic optimization. We evaluated 16 CGMMV primer combinations, identifying CGMMV-F1/CGMMV-R3 as optimal due to distinct amplification bands in target reactions and absence of non-specific amplification in non-template controls (Fig. S22). Evaluation of three partzyme pairs revealed CGMMV-P A3/B3 yielded maximal fluorescence intensity and F/F₀ values (Fig. S23) and was selected for subsequent assays.

Specificity assessment confirmed significant fluorescence signals exclusively in CGMMV-positive samples, with no cross-reactivity observed in other plant viruses (Fig. S24). Comparative analysis of 13 watermelon seed samples demonstrated complete diagnostic concordance (100%) between OAR-MNA and qPCR methods (Figs. S25, S26), validating the platform's reliability for field-deployable detection of RNA viral pathogens.


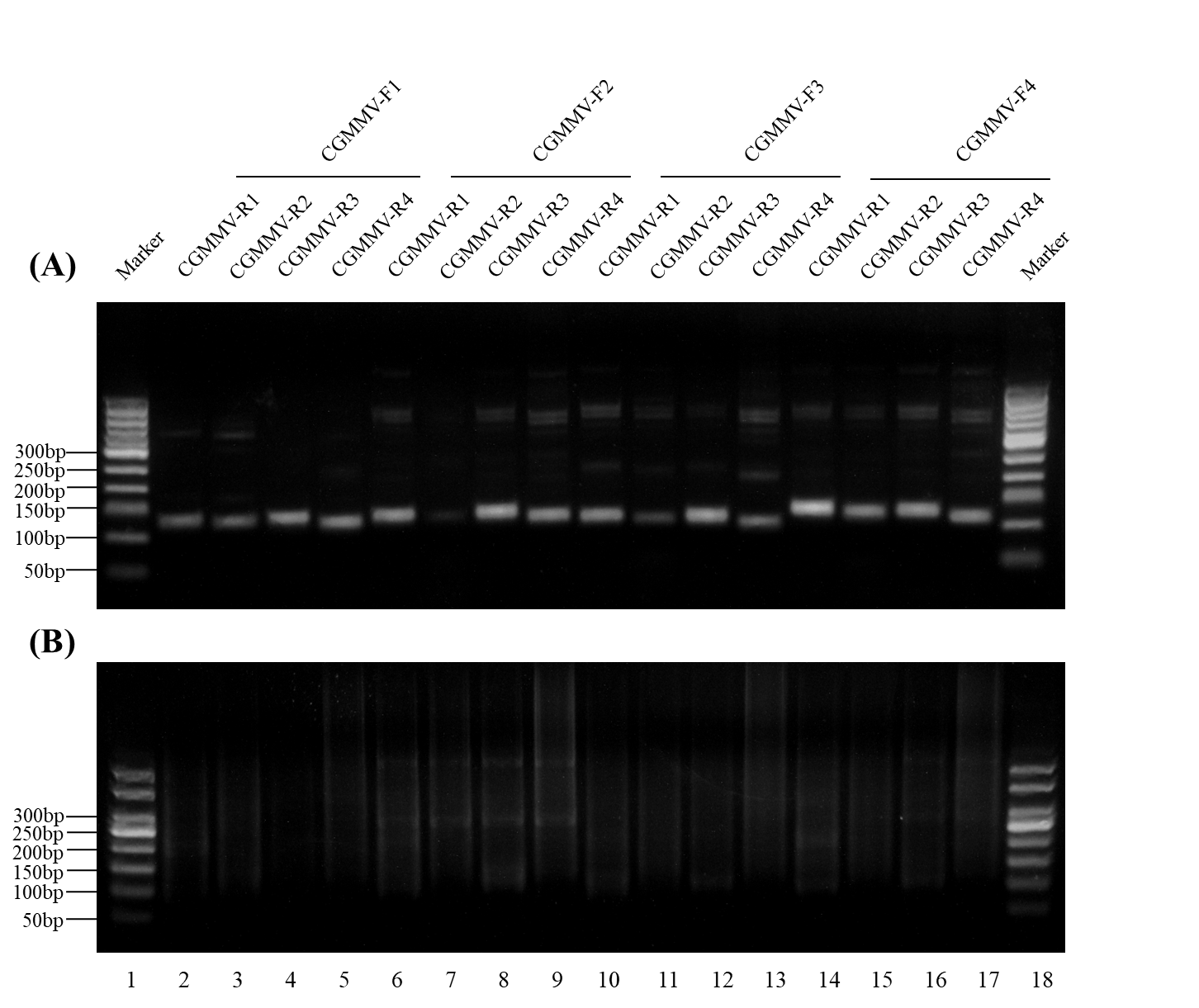


**Figure S22 Electrophoresis of RT-RPA amplicon with CGMMV RPA primer pairs.** (A) with templates and (B) without templates. Bands were visualized with the Bio-Rad XR+ imaging system, alongside a 50-500 bp DNA ladder.


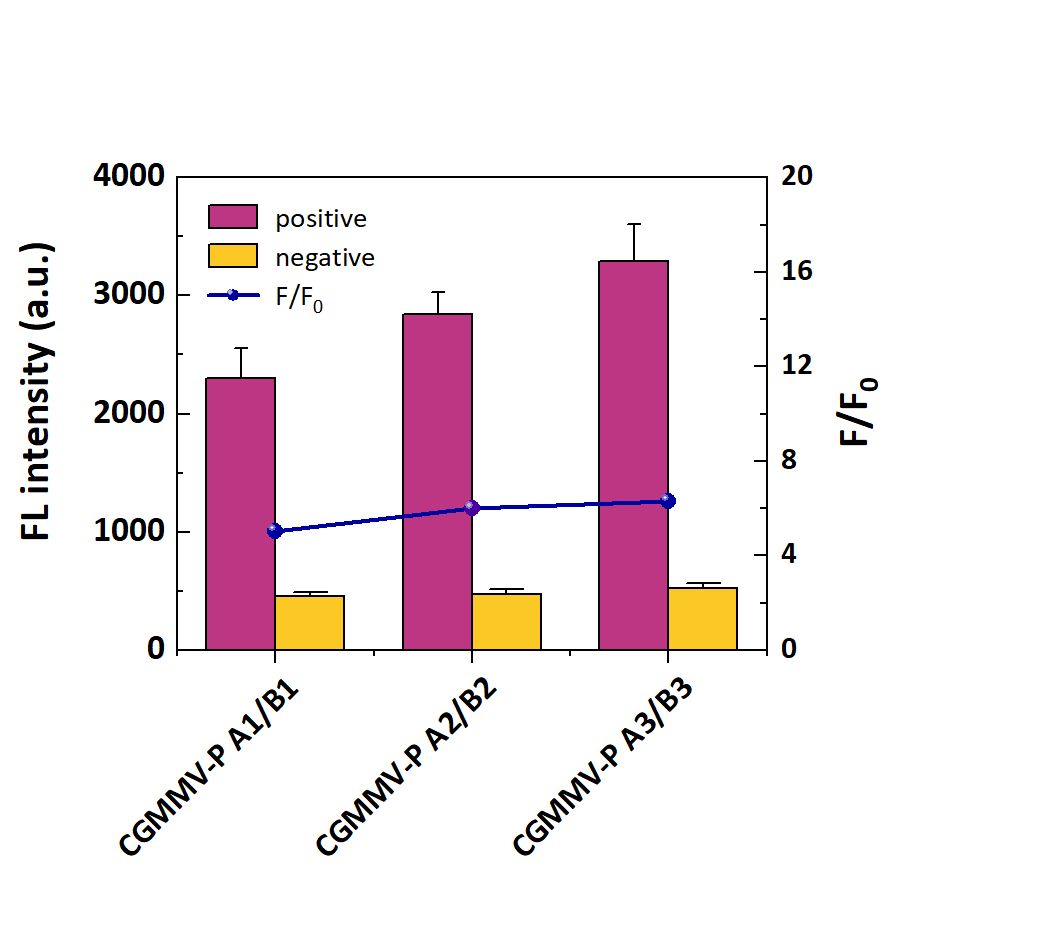


**Figure S23. Optimization of CGMMV-specific partzyme pairs.** Fluorescence signals of three CGMMV-P A/B variants. Error bars present means ± standard deviation from triplicate experiments. F: Fluorescence intensity with target; F₀: Background fluorescence without target.


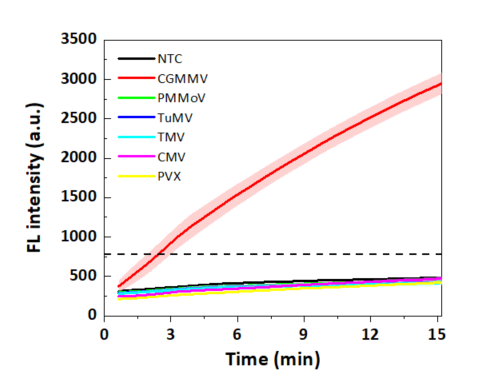


**Figure S24. Specificity validation of OAR-MNA for CGMMV detection.** Real-time detection curves among six plant viruses: Cucumber green mottle mosaic virus (CGMMV), Cucumber mosaic virus (CMV), Potato virus X (PVX), Turnip mosaic virus (TuMV), Pepper mild mottle virus (PMMoV), and Tobacco mosaic virus (TMV). NTC: Non-template control. Dashed line: threshold fluorescence intensity.


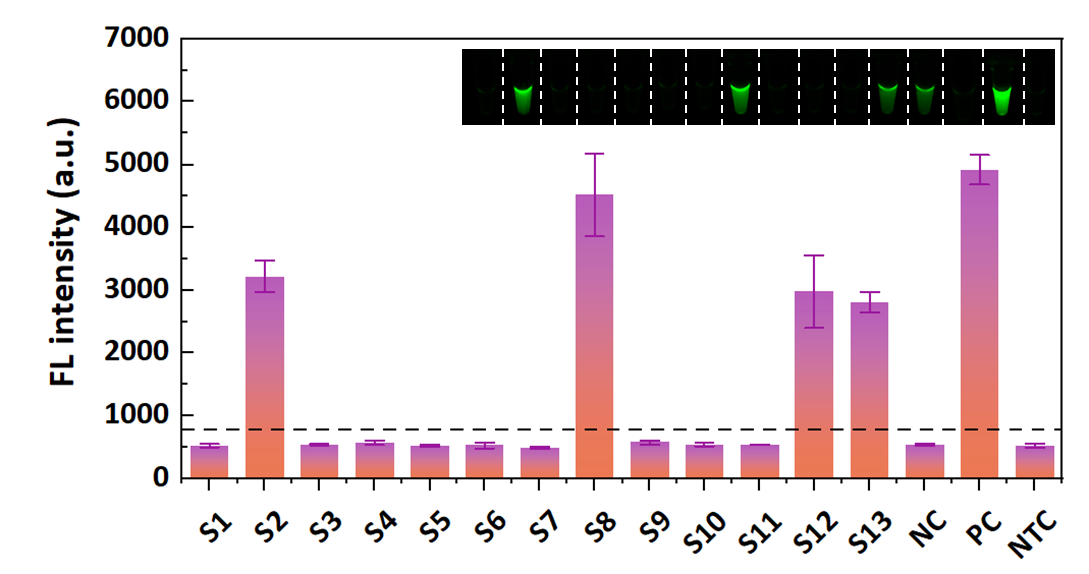


**Figure S25. Field detection of CGMMV in watermelon seeds using OAR-MNA.** Fluorescence signals and endpoint images for 13 seed samples. Error bars present means ± standard deviation from triplicate experiments. NC: Negative control; PC: Positive control; NTC: Non-template control. Dashed line: Threshold fluorescence intensity.


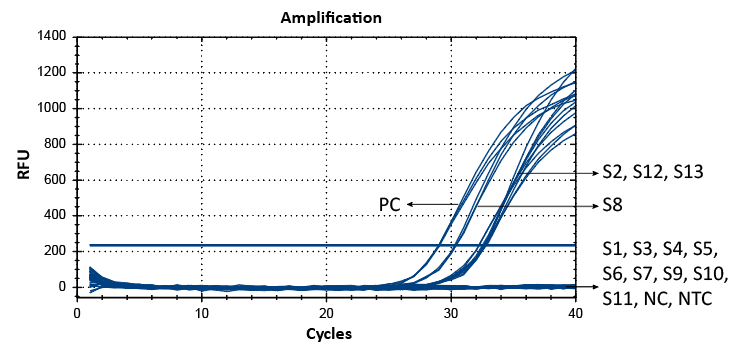


**Figure S26. qPCR validation of CGMMV in watermelon seed samples.** Real-time curves corresponding to samples in Figure S25. NC: Negative control; PC: Positive control; NTC: Non-template control. All samples tested in triplicate.


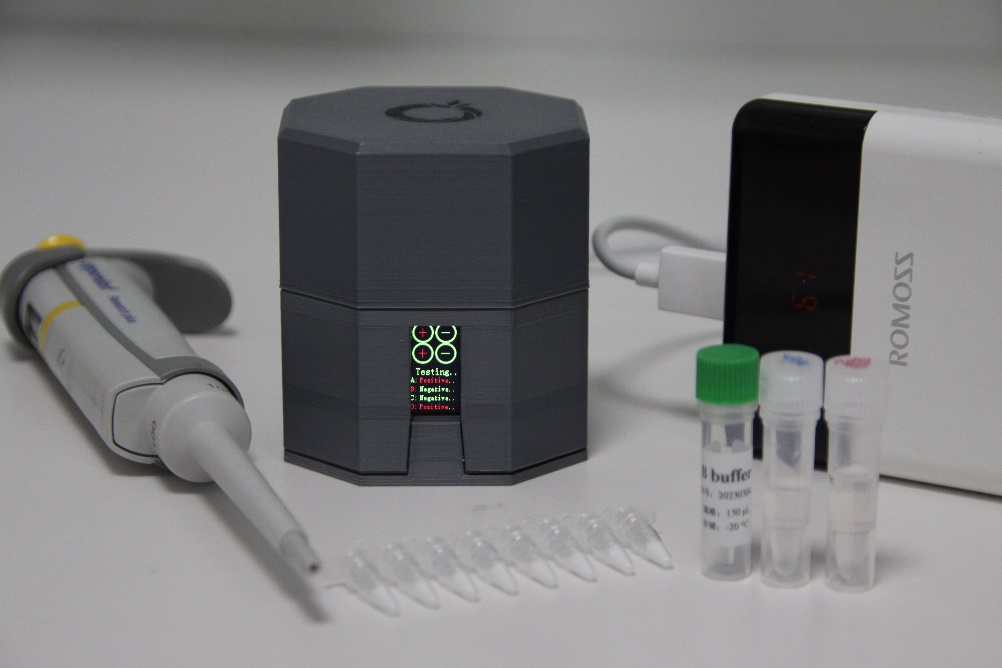


**Figure S27. Detection of samples by the OAR-MNA biosensor using a DNA analyzer in a resource-limited field environment.** The equipment and reagents required for the experiment include a pocket-size DNA analyzer, a power bank to supply electricity to the DNA analyzer, a pipette, freeze-dried reagent microspheres, and the corresponding buffer.

**Table S1. Sequences of primers and probes used in this work.**

| Item | Oligo name | Sequence (5’-3’) |
| --- | --- | --- |
| Conventional PCR primer | CGMMV-SP1 | CGTGGTAAGCGGCATTCTAAACCTC |
|  | CGMMV-T7-SP1 | GAAATTAATACGACTCACTATAGGGCGTGGTAAGCGGCATTCTAAACCTC |
|  | CGMMV-AP1 | CCGCAAACCAATGAGCAAACCG |
| qPCR primer and probe | AC-FP | CTGATAATCCTCGGCTCAACAA |
|  | AC-RP | TGAGCGCATTTCTGACGAG |
|  | AC-qP | [FAM]AAGAAATACGCCCTCGCCAATCTCC[BHQ1] |
|  | CGMMV-F | GCATAGTGCTTTCCCGTTCAC |
|  | CGMMV-R | TGCAGAATTACTGCCCATAGAAAC |
|  | CGMMV-P | [FAM]CGGTTTGCTCATTGGTTTGCGGA[BHQ1] |
| RPA primer and probe | AC-SP-2 | TCACCGGCGGCACGGTGCAGTTTCCTGCTT |
|  | AC-AP-2 | CCAATACGCTCTGCGGTAGGGCGAAGAAAC |
|  | Ac-F3 | gttttgacgcaatcaaatttttgtcaccgg |
|  | Ac-R2 | CGGCTTCGCGAGAGGCCTCTTTGTTGTTGG |
|  | Ac-P | TTCCGCCGGCAACGCTGATTCGACTCTA[FAM-dT]G[THF]A[iBHQ1-dT]TTTTAAAGAACAG[3’-block] |
|  | CGMMV-F1 | TTTACGATAGGGCTTCATTTGAAGCCGCGT |
|  | CGMMV-F2 | ACGATAGGGCTTCATTTGAAGCCGCGTTTT |
|  | CGMMV-F3 | TTGAAGCCGCGTTTTCGGTAGTCTGGTCAG |
|  | CGMMV-F4 | TACGATAGGGCTTCATTTGAAGCCGCGTTT |
|  | CGMMV-R1 | GTGAACGGGAAAGCACTATGCACTTTGGTG |
|  | CGMMV-R2 | GGGAAAGCACTATGCACTTTGGTGTGCACC |
|  | CGMMV-R3 | TGAACGGGAAAGCACTATGCACTTTGGTGT |
|  | CGMMV-R4 | GCACTATGCACTTTGGTGTGCACCACCATC |
| Sequencing primer | SP-2X | TCACCGGCGGCACGGTGC |
|  | AP-2X | CCAATACGCTCTGCGGTAG |
|  | F3-X | GTTTTGACGCAATCAAATT |
|  | R2-X | CGGCTTCGCGAGAGGCCTC |
| MNAzyme partzyme and probe | AC-P A1 | ATTCATAGAGTCGAAACAACGAGATGAATACTT |
|  | AC-P B1 | ATCTGACGGAGGCTAGCTTCAGCGTTGCCGG |
|  | AC-P A2 | CATAGAGTCGAAACAACGAGATGAATACTT |
|  | AC-P B2 | ATCTGACGGAGGCTAGCTTCAGCGTTGCC |
|  | AC-P A3 | AAATTCATAGAGTCGAAACAACGAGATGAATACTT |
|  | AC-P B3 | ATCTGACGGAGGCTAGCTTCAGCGTTGCCGGCGG |
|  | CGMMV-P A1 | CTTTGGTGTGCAACAACGAGATGAATACTT |
|  | CGMMV-P B1 | ATCTGACGGAGGCTAGCTCCACCATCAGA |
|  | CGMMV-P A2 | ACTTTGGTGTGCAACAACGAGATGAATACTT |
|  | CGMMV-P B2 | ATCTGACGGAGGCTAGCTCCACCATCAGAA |
|  | CGMMV-P A3 | GCACTTTGGTGTGCAACAACGAGATGAATACTT |
|  | CGMMV-P B3 | ATCTGACGGAGGCTAGCTCCACCATCAGAAGA |
|  | MNA FQ-probe | [FAM]CCACCACGAGTATTCATC/rG//rU/CCGTCAGACGTGGTGG [BHQ1] |
| ssDNA target | AC-ssDNA | GGCACGGTGCAGTTTCCTGCTTTCCGCCGGCAACGCTGATTCGACTCTATGAATTTTTAAAGAACAGCCGATTGATCCGG |
|  | AC-ssDNA1 | GTTTCCTGCTTTCCGCCGGCAACGCTGATTCGACTCTATGAATTTTTAAAGAACAGCCG |
|  | AA-ssDNA1 | GTTTCCTGCTTTCCGCCGGCAACGCAGATTAGACTCTATGAATTTTTAAAGAACAGCCG |

**Table S2. Comparison of fluorescent intensities between pure *Acidovorax citrulli* cultures and *A. citrulli*-spiked watermelon homogenates at different concentrations.**

|  | qPCR | | OAR-MNA biosensor | |
| --- | --- | --- | --- | --- |
|  | Pure bacterial colony | Spiked watermelon homogenate | Pure bacterial colony | Spiked watermelon homogenate |
|  | Ct value | Ct value | FL intensity | FL intensity |
| 0 (CFU/mL) | >40 | >40 | 712.6±11.25^1^ | 734.13±29.10 |
| 2.8x10^2^ (CFU/mL) | 38.30±0.23 | 38.85±0.62 | 1459.98±70.00 | 1217.63±20.95 |
| 2.8x10^4^ (CFU/mL) | 33.64±0.12 | 33.98±0.10 | 3751.74±56.16 | 3032.84±109.98 |
| 2.8x10^6^ (CFU/mL) | 28.67±0.17 | 28.46±0.07 | 4337.98±53.23 | 4343.66±46.53 |

^1^ The positive threshold value of OAR-MNA biosensor in BFB detection is 907.5

**Table S3. Comparison of the working principle, key advantages, disadvantages and costs between the most widely used nucleic acid-based detection methods in plant disease detection and our established method.**

| Method | Working principle | Advantages | Disadvantages | Cost ($)^1^ | Ref |
| --- | --- | --- | --- | --- | --- |
| PCR-based methods | PCR-based amplification in a thermal cycler | High sensitivity and specificity | Requires thermal cycling instruments | $0.93 (qPCR) $0.22 (PCR) | Bustin, 2002; Huggett et al., 2013 |
| LAMP | Amplification at 60-65 ℃with four to six primers | High sensitivity, Thermal stability | Hard for multiplexing, Complicated primer design | $3.72 | Notomi et al., 2000; Notomi et al., 2015 |
| RCA | Form a long ssDNA/ RNA by a circular DNA template | Readily multiplexed; low cost | Labor-intensive operation; Time consuming | $2.76 | Ali et al., 2014; Zhao et al., 2008 |
| RPA | Amplification at 37-42 ℃ | Extremely fast; Anti-interference; Low work TEMP | Nonspecific amplification; High cost | $4.69 | Magrina Lobato & O'Sullivan, 2018; Munawar, 2022 |
| RPA combined CRISPR/Cas assay | RPA combined with CRISPR/Cas system | High sensitivity and specificity; | Requires cold chain and skilled design; | $3.21 | Kellner et al., 2019 |
| Nucleic Acid Aptamers based detection | ssDNA that efficiently catalyzes the cleavage | Low cost; Enzyme-free Quantifiable | Labor-intensive operation; Time consuming | $0.48 | Cho et al., 2009; Yu et al., 2021 |
| OAR-MNA Biosensor | Asymmetric RPA combined with MNAzyme | High sensitivity and specificity; Thermal stability; Low work TEMP | multiplexing has not been established | $2.18 | This study |

^1^ To ensure a fair and accurate cost comparison, all reagent quotations were sourced from reputable Chinese companies in March 2025. RPA kits were sourced from Amp-Future Biotech Co., Ltd. (Changzhou, China), LAMP kits from LMAI Bio Co., Ltd. (Shanghai, China), and RCA kits from Shanghai Zeye Biotechnology Co., Ltd. (Shanghai, China).For more complex or combined methods, such as RPA-CRISPR/Cas and RPA-MNAzyme, where commercial kits are not readily available, we calculated costs based on the individual reagent quantities reported in relevant published studies and the quotation for company. Nucleic acid components (primers, gRNA, probes) and CRISPR/Cas proteins were procured from Sangon Biotech (Shanghai, China) and Tolo Biotech (Shanghai, China), respectively.

**Table S4. Comparison of the sensitivity, specificity and procedures between the most widely used nucleic acid-based detection methods in plant disease detection and our established method.**

| Method | Details^1^ | Targeted plant pathogens | Sensitivity^2^ | Specificity | Procedure | Ref |
| --- | --- | --- | --- | --- | --- | --- |
| PCR-based methods | ddPCR | *Acidovorax citrulli* | 20 fg/μL genome (4 copies/μL) | not referred | 95 ℃, 10 min; (94 ℃, 30 sec; 60 ℃, 6 sec) 45 cycles; 98 ℃, 10 min | Lu et al., 2020 |
|  | qPCR | *Colletotrichum theobromicola* | 1.4pg/μL genome (23 copies/μL) | 100% | 95 ℃, 2 min; (95 ℃,15 sec; 63.5 ℃, 30 sec) 40 cycles | Kaur et al., 2021 |
| LAMP | / | *Acidovorax citrulli* | 1 pg/μL genome (200 copies/μL) | 100% | 64 ℃, 60 min | Yan et al., 2019 |
|  | / | *Colletotrichum acutatum* | 0.8-8 pg/μL genome (12-120 copies/μL) | 100% | 63 ℃, 50 min | Zhang et al., 2016 |
| RCA | Cas12-RCA | *Alternaria* species | 22 pM dsDNA (>5000 copies/μL) | 100% | 45 ℃, 40 min; 25 ℃, 45 min; 37 ℃, 30 min; 42 ℃, 40 min; 25 ℃, 10 min | Ma et al., 2024 |
|  | RPA/Cas12-RCA | *Alternaria* species | 0.3072 fg/μL plasmid^3^ | 100% | 37 ℃, 25 min; 37 ℃, 30 min; 37 ℃, 30 min; 42 ℃, 30 min; 37 ℃, 10 min | Liu et al., 2023 |
| RPA | / | *Leptosphaeria maculans* | 219 copies/μL | not referred | 39 ℃, 30 min | Lei et al., 2019 |
|  | RPA-LFD | *Candidatus* Phytoplasma mali | 10 copies/μL | 100% | 42 ℃, 20 min | Valasevich & Schneider, 2017 |
| RPA combined CRISPR/Cas assay | / | *Phytophthora cambivora* | 10 pg/μL genome (110 copies/μL) | 100% | 37 ℃, 15 min; 37 ℃, 15 min | Li et al., 2024 |
|  | RPA/Cas12a-LFD | *Diaporthe aspalathi* and *Diaporthe caulivora* | 14.5 copies/μL *D. aspalathi;* 24.6 copies/μL *D. caulivora* | 100% | 39 ℃, 20 min; 37 ℃, 30 min | Sun et al., 2024 |
| Nucleic Acid Aptamers based detection | SDA-DNAzyme | Sugarcane smut fungus | 56.76 aM plasmid (35 copies/μL) | not referred | - ℃, 120 min; - ℃, 50 min; - ℃, 80 min; - ℃, 120 min; | Che et al., 2025 |
|  | DNAzyme | Cucumber green mottle mosaic virus | 0.91μg/mL ssDNA (>5000 copies/μL) | 100% | 37 ℃, 30 min; 37 ℃,30 min; 37 ℃, 15 min; 85 ℃, 5 min; - ℃, 80 min; 37 ℃, 15 min | Wang et al., 2019 |
| OAR-MNA Biosensor | aRPA-MNAzyme | *Acidovorax citrulli* | 20 copies/μL | 100% | 41 ℃, 15 min; 41 ℃, 30 min; | This study |

^1^ For studies employing multiple detection methods in combination, all methodological components are listed on this column.

^2^All sensitivity values have been standardized to copies/μL (shown in brackets) to enable direct cross-study comparison. The conversion was calculated using: copies/μL = (C × Nₐ)/ (L × 660), where C is mass concentration (g/μL), Nₐ is Avogadro's number (6.022×10²³ molecules/mol), and L is target length (bp).

^3^ Due to the lack of plasmid information used in this study, the target sequence length is unknown, and the copy number could not be calculated.

**References**

Ali, M.M., Li, F., Zhang, Z., Zhang, K., Kang, D.-K., Ankrum, J.A., Le, X.C. and Zhao, W. (2014) Rolling circle amplification: a versatile tool for chemical biology, materials science and medicine. Chem. Soc. Rev., 43, 3324-3341.

Bustin, S.A. (2002) Quantification of mRNA using real-time reverse transcription PCR (RT-PCR): trends and problems. J. Mol. Endocrinol., 29, 23-29.

Che, R.S., Tang, D.Y., Fu, B.T., Yan, F.Y., Yan, M.X., Wu, Y.Y., Yan, J., Huang, K.J., Ya, Y. and Tan, X.C. (2025) Dual-modal improved biosensing platform for sugarcane smut pathogen based on biological enzyme-Mg^2+^ DNAzyme coupled with DNA transporter cascading hybridization chain reaction. Int. J. Biol. Macromol., 286, 138403.

Cho, E.J., Lee, J. W. and Ellington, A.D. (2009) Applications of aptamers as aensors. Annu. Rev. Anal. Chem., 2, 241-264.

Ding, L., Wang, X., Chen, X., Xu, X., Wei, W., Yang, L., Ji, Y., Wu, J., Xu, J., Peng, C., 2024. Development of a novel Cas13a/Cas12a-mediated 'one-pot' dual detection assay for genetically modified crops. J. Adv. Res., <https://doi.org/10.1016/j.jare.2024.07.027>.

Huggett, J.F., Foy, C.A., Benes, V., Emslie, K., Garson, J.A., Haynes, R., Hellemans, J., Kubista, M., Mueller, R.D., Nolan, T., Pfaffl, M.W., Shipley, G.L., Vandesompele, J., Wittwer, C.T. and Bustin, S.A. (2013) The digital MIQE guidelines: Minimum information for publication of quantitative digital PCR experiments. Clin. Chem., 59, 892-902.

Kaur, H., Singh, R., Doyle, V. and Valverde, R. (2021) A diagnostic TaqMan real-time PCR assay for in planta detection and quantification of *Colletotrichum theobromicola*, causal agent of boxwood dieback. Plant Dis., 105, 2395-2401.

Kellner, M.J., Koob, J.G., Gootenberg, J.S., Abudayyeh, O.O. and Zhang, F. (2019) SHERLOCK: nucleic acid detection with CRISPR nucleases. Nat. Protoc., 14, 2986-3012.

Lei, R., Kong, J., Qiu, Y., Chen, N., Zhu, S., Wang, X. and Wu, P. (2019) Rapid detection of the pathogenic fungi causing blackleg of *Brassica napus* using a portable real-time fluorescence detector. Food Chem., 288, 57-67.

Li, Z., Feng, W., Zhu, Z., Lu, S., Lin, M., Dong, J., Wang, Z., Liu, F. and Chen, Q. (2024) Cas-OPRAD: a one-pot RPA/PCR CRISPR/Cas12 assay for on-site *Phytophthora* root rot detection. Front. Microbiol., 15, 1390422.

Liu, Y., Ma, L., Liu, W., Xie, L., Wu, Q., Wang, Y., Zhou, Y., Zhang, Y., Jiao, B. and He, Y. (2023) RPA-CRISPR/Cas12a combined with rolling circle amplification- enriched DNAzyme: A homogeneous photothermal sensing strategy for plant pathogens. J. Agr. Food Chem., 71, 4736-4744.

Lu, Y., Zhang, H.-J., Zhao, Z.-J., Wen, C.-L., Wu, P., Song, S.-H., Yu, S.-C., Luo, L.-X. and Xu, X.-L. (2020) Application of droplet digital PCR in detection of seed-transmitted pathogen *Acidovorax citrulli*. J. Integr. Agr., 19, 561-569.

Ma, L., Xie, L., Wu, Q., Yang, L., Zhou, Y., Cui, Y., Zhang, Y., Jiao, B., Wang, C. and He, Y. (2024) Integrating CRISPR-Cas12a and rolling circle-amplified G-quadruplex for naked-eye fluorescent "off-on" detection of citrus *Alternaria*. Int. J. Biol. Macromol., 262, 129983.

Magrina Lobato, I. and O'sullivan, C.K. (2018) Recombinase polymerase amplification: Basics, applications and recent advances. Trac-Trends Anal Chem., 98, 19-35.

Munawar, M.A. (2022) Critical insight into recombinase polymerase amplification technology. Expert Rev. Mol. Diagn., 22, 725-737.

Notomi, T., Mori, Y., Tomita, N. and Kanda, H. (2015) Loop-mediated isothermal amplification (LAMP): principle, features, and future prospects. J.Microbiol., 53, 1-5.

Notomi, T., Okayama, H., Masubuchi, H., Yonekawa, T., Watanabe, K., Amino, N. and Hase, T. (2000) Loop-mediated isothermal amplification of DNA. Nucleic Acids Res., 28, e63.

Sun, X., Lei, R., Zhang, H., Chen, W., Jia, Q., Guo, X., Zhang, Y., Wu, P. and Wang, X. (2024) Rapid and sensitive detection of two fungal pathogens in soybeans using the recombinase polymerase amplification/CRISPR-Cas12a method for potential on-site disease diagnosis. Pest Manag. Sci., 80, 1168-1181.

Valasevich, N. and Schneider, B. (2017) Rapid detection of "*Candidatus* Phytoplasma mali" by recombinase polymerase amplification assays. J. Phytopathol., 165, 762-770.

Wang, C., Wang, X., Wei, W., Chen, X., Shen, J., Xu, J. and Cai, J. (2022) Establishment of on-site rapid dual-mode fluorescence RPA detection method for bacterial-fruit of blotch. Acta Agriculturae Zhejiangensis, 34, 1519-1528.

Wang, Y., Liu, J. and Zhou, H. (2019) Visual detection of cucumber green mottle mosaic virus based on terminal deoxynucleotidyl transferase coupled with dnazymes amplification. Sensors, 19, 1298

Yan, L., Zhao, Y., Zhou, J., Chen, S., Bai, S., Tian, Y., Gong, W. and Hu, B. (2019) Rapid and sensitive detection of *Acidovorax citrulli* in cucurbit seeds by visual loop-mediated isothermal amplification assay. J. Phytopathol., 167, 10-18.

Yang, L., Chen, G., Wu, J., Wei, W., Peng, C., Ding, L., Chen, X., Xu, X., Wang, X. and Xu, J. (2024) A PAM-free one-step asymmetric RPA and CRISPR/Cas12b combined assay (OAR-CRISPR) for rapid and ultrasensitive DNA detection. Anal. Chem., 96, 5471-5477.

Yu, H., Alkhamis, O., Canoura, J., Liu, Y. and Xiao, Y. (2021) Advances and challenges in small-molecule DNA aptamer isolation, characterization, and sensor development. Angew. Chem. Int. Edit., 60, 16800-16823.

Zhang, X., Harrington, T.C., Batzer, J.C., Kubota, R., Peres, N.A. and Gleason, M.L. (2016) Detection of *Colletotrichum acutatum* sensu lato on strawberry by loop-mediated isothermal amplification. Plant Dis., 100, 1804-1812.

Zhao, W., Ali, M.M., Brook, M.A. and Li, Y. (2008) Rolling circle amplification: Applications in nanotechnology and biodetection with functional nucleic acids. Angew. Chem. Int. Edit., 47, 6330-6337.
